# Supplementary material for: Genetics-informed precision treatment formulation in schizophrenia and bipolar disorder
Source: Am J Hum Genet. 2022 Sep 1;109(9):1620–37. doi: 10.1016/j.ajhg.2022.07.011 (PMC9502060; doi:10.1016/j.ajhg.2022.07.011)
Supplement: Document S4. Article plus supplemental information [file mmc4.pdf]

ARTICLE

Genetics-informed precision treatment formulation in schizophrenia and bipolar disorder

Authors

William R. Reay, Michael P. Geaghan, Joshua R. Atkins, Vaughan J. Carr, Melissa J. Green, Murray J. Cairns

Correspondence

murray.cairns@newcastle.edu.au

Reay et al. propose an approach to facilitate precision treatment using genetics. Drug targets are first prioritized through integrating genetics with expression data. These compounds could then be directed more specifically to individuals based on genetic risk annotated to the network of genes that interact with prioritized targets.

Reay et al., 2022, The American Journal of Human Genetics *109*, 1620–1637
September 1, 2022

© 2022 The Author(s).

https://doi.org/10.1016/j.ajhg.2022.07.011

ARTICLE

Genetics-informed precision treatment formulation in schizophrenia and bipolar disorder

William R. Reay,[1,2] Michael P. Geaghan,[3] Joshua R. Atkins,[1] Vaughan J. Carr,[4,5,6] Melissa J. Green,[4,5] and Murray J. Cairns[1,2,*]

Summary

Genetically informed drug development and repurposing is an attractive prospect for improving patient outcomes in psychiatry; however, the effectiveness of these endeavors is confounded by heterogeneity. We propose an approach that links interventions implicated by disorder-associated genetic risk, at the population level, to a framework that can target these compounds to individuals. Specifically, results from genome-wide association studies are integrated with expression data to prioritize individual "directional anchor" genes for which the predicted risk-increasing direction of expression could be counteracted by an existing drug. While these compounds represent plausible therapeutic candidates, they are not likely to be equally efficacious for all individuals. To account for this heterogeneity, we constructed polygenic scores restricted to variants annotated to the network of genes that interact with each directional anchor gene. These metrics, which we call a pharmagenic enrichment score (PES), identify individuals with a higher burden of genetic risk, localized in biological processes related to the candidate drug target, to inform precision drug repurposing. We used this approach to investigate schizophrenia and bipolar disorder and reveal several compounds targeting specific directional anchor genes that could be plausibly repurposed. These genetic risk scores, mapped to the networks associated with target genes, revealed biological insights that cannot be observed in undifferentiated genome-wide polygenic risk score (PRS). For example, an enrichment of these partitioned scores in schizophrenia cases with otherwise low PRS. In summary, genetic risk could be used more specifically to direct drug repurposing candidates that target particular genes implicated in psychiatric and other complex disorders.

Introduction

Psychiatric disorders remain difficult to effectively manage in some patients, with treatment resistance observed in a notable proportion of individuals prescribed conventional pharmacotherapies.[1–3] Moreover, a key challenge in psychiatric practice is the selection of a suitable course of treatment for newly diagnosed patients. Novel treatment opportunities for these disorders would be of great clinical benefit, but the drug development pipeline remains arduous, expensive, and unproductive.[4,5] Drug repurposing, whereby an approved compound is redeployed for a new indication, is a promising avenue to more rapidly alter psychiatric practice relative to the *de novo* drug development process.[6,7] There has already been utility in this approach demonstrated in psychiatry, such as atomoxetine that has been repurposed for attention deficit hyperactive disorder (ADHD [MIM: 143465]) and the anti-convulsant valproate for bipolar disorder.[8]

We know that psychiatric illnesses arise from a multifaceted interplay between genetic and environmental factors that contribute to its etiologic complexity. In recent years, genome-wide association studies (GWASs) have confirmed that psychiatric disorders are polygenic in nature,[9–13] with common frequency variants constituting a significant portion of trait heritability. This means that individual loci that have small to modest impact, but contribute to a much larger polygenic effect of many such variants throughout the genome.[14,15] Biological insights from genetic studies could lead to repurposing opportunities in psychiatry—for example, schizophrenia GWASs have previously suggested that the common variant signal is enriched among the targets of antiepileptics, as well as in genes involved in retinoid (vitamin A derivative) pathways.[16,17] The polygenic nature of these disorders, however, present a challenge for drug targeting because the genetic architecture of each individual will be highly heterogeneous. This means that any given patient will carry a unique combination of risk and protective alleles, which likely translates to different underlying biological processes being affected. As a result, genetically informed drug candidates may not be efficacious at a population level. These phenomena necessitate the consideration of how pharmacotherapies could be targeted more specifically to individuals based on their underlying genetic and clinical risk factors.

Our group has previously sought to address these challenges through the development of the pharmagenic enrichment score (PES), which is a framework that seeks to use polygenic risk to direct precision drug repurposing

[1]Centre for Complex Disease and Precision Medicine, School of Biomedical Sciences and Pharmacy, The University of Newcastle, Callaghan, NSW, Australia; [2]Precision Medicine Research Program, Hunter Medical Research Institute, Newcastle, NSW, Australia; [3]Kinghorn Centre for Clinical Genomics, Garvan Medical Research Institute, Darlinghurst, NSW, Australia; [4]Discipline of Psychiatry and Mental Health, School of Clinical Medicine, University of New South Wales, Randwick, NSW, Australia; [5]Neuroscience Research Australia, Sydney, NSW, Australia; [6]Department of Psychiatry, Monash University, Melbourne, VIC, Australia

*Correspondence: murray.cairns@newcastle.edu.au
https://doi.org/10.1016/j.ajhg.2022.07.011.
© 2022 The Author(s). This is an open access article under the CC BY license (http://creativecommons.org/licenses/by/4.0/).

opportunities.[7,18–20] Specifically, the PES approach derives partitioned polygenic scores from variants annotated to pathways or networks that are targeted by approved drugs, with the underlying hypothesis that individuals with elevated genetic risk (PES) among those genes may benefit from a compound which modulates that pathway. Furthermore, in prior work we have established that PES profiles provide distinct insights from a biologically undifferentiated genome-wide polygenic risk score (PRS).[18–20] However, a limitation of the PES approach is that it is not innately informative as to which of the suite of drugs targeting a pathway will be most useful, particularly in regards as to whether an agonist or antagonist of target genes should be investigated. While we addressed this previously, in respiratory medicine, by triangulating prioritized pathways through causal inference of pharmacologically sensitive biochemical traits,[19] these relationships are more difficult to find in psychiatric disorders.[21] In the current study, we propose a new implementation of the PES that is informed by genetically proxied mRNA or protein expression of drug target genes. This approach identifies candidate psychiatric drug repurposing opportunities at the population level that can then be more appropriately integrated with genetic risk scores relevant to these target genes, to identify individuals who may benefit more readily from these compounds. These are termed directional anchor genes as they inform on the clinically useful direction of modulation is for the biological networks containing these genes which can be utilized to construct PESs. In this study, we investigated this novel approach in two highly heritable psychiatric disorders, schizophrenia (MIM: 181500) and bipolar disorder, identified novel drug repurposing opportunities from candidate directional anchor genes, and propose how these genes could be used in concert with the PES to direct the candidate compounds for repurposing.

Material and methods

Overview of the directional anchor gene pharmagenic enrichment score approach

We summarize the fundamental principles of directional anchor genes and their integration with the pharmagenic enrichment score in this section, followed by more expansive details in the subsequent sections. In brief, we define the concept of a directional anchor gene (DA-gene) as a gene where (1) the direction of expression associated with increased odds of the disorder can be predicted and (2) this disorder-associated direction of expression could be counteracted by an approved compound, thus constituting a drug repurposing opportunity. For instance, if upregulation of a hypothetical gene, gene *X*, was associated with greater odds of a disease phenotype, then an antagonist of gene *X* may be clinically useful. If this gene *X* antagonist is already approved for another indication, this may inform drug repurposing. However, there is immense heterogeneity between individuals for any given complex trait or disease in its genetic architecture, which often translates to highly variable clinical manifestation. We therefore hypothesize that individuals with a greater burden of disorder-associated polygenic risk in the directional anchor gene, and its network of genes that physically and biologically interact with it, may benefit more specifically from a drug repurposing candidate targeting the DA gene. Polygenic risk mapped to biological networks encompassing the directional anchor genes is likely to incorporate disorder-associated impacts on upstream processes that would modify the effect of a compound targeting the candidate gene, as well as downstream processes triggered by modulating the directional anchor. As discussed in the introduction, our group has previously developed the pharmagenic enrichment score (PES) methodology to utilize polygenic scoring to direct drug repurposing, whereby polygenic scores are constructed specifically using variants mapped to biological pathways targeted by known drugs.[18,19] A limitation of the PES approach is that it does not inherently predict the direction of effect genes in the pathway that would need to be targeted such that repurposing a drug for individuals with high polygenic risk in said pathway would be efficacious. Directional anchor genes, therefore, help address this limitation when used in conjunction with PES constructed using networks or biological pathways in which the gene participates. In other words, drug repurposing opportunities are predicted at the population level based on the expression of target genes, with these compounds potentially able to be more specifically directed to individuals with elevated disorder-associated genetic risk within pathways or networks that contain the directional anchor gene, that is, an elevated PES (Figure 1). It should be noted that while we apply this approach to binary disease phenotypes in this study, it can also be utilized for clinically relevant continuous traits. In that case, candidate directional anchor genes would be those genes for which the drug repurposing candidate is genetically inferred to modulate the trait in a clinically useful fashion.

Schizophrenia and bipolar disorder genome-wide association studies

We obtained GWAS summary statistics for schizophrenia (SZ) and bipolar disorder (BIP) from the psychiatric genomics consortium.[9,10] The SZ GWAS was a mega-analysis of mostly European ancestry cohorts and comprised 67,390 affected individuals and 94,015 control subjects, while the European ancestry BIP GWAS mega-analysis had 20,352 affected individuals and 31,358 control subjects. In addition, we also utilized the same SZ GWAS with a constituent cohort removed (Australian Schizophrenia Research Bank) when we profiled PES within that dataset, as described in Training and validation of directional anchor gene network PES.

Transcriptome and proteome-wide association studies

A transcriptome-wide association study (TWAS) and a proteome-wide association study (PWAS) was performed of SZ and BIP by leveraging genetically imputed models of mRNA and protein expression, respectively. Specifically, we utilized the FUSION approach for TWAS/PWAS, with full details outlined in the supplemental material and methods.[22] Expression weights for the TWAS were derived from post-mortem brain (GTEx v7, PsychENCODE) and whole blood (GTEx v7), while protein expression weights were similarly from post-mortem brain (ROSMAP) and whole blood (ARIC).[22–25] The FUSION methodology integrates SNP effects from the model of genetically predicted expression with the effects of the same SNPs on SZ or BIP, after accounting for linkage disequilibrium, such that the TWAS *Z* score can be a conceptualized measure of genetic

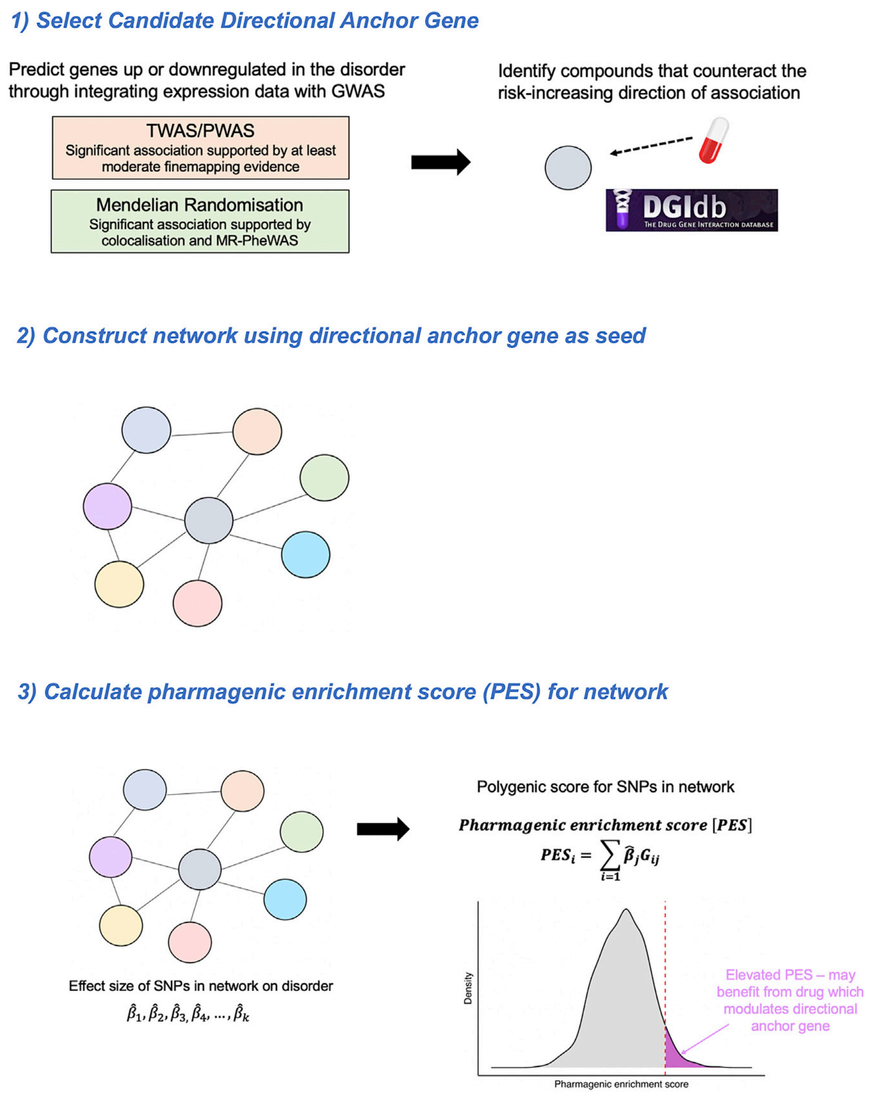

Figure 1. Overview schematic of the integration of candidate directional anchor genes with pharmagenic enrichment scores
Directional anchor genes are genes targeted by an approved compound, in what is genetically predicted (through integration with expression data) to decrease the risk of the disorder or modulate the trait in a clinically useful manner. For instance, if increased expression of a gene is associated with a disorder through a transcriptome or proteome-wide association study (TWAS/PWAS) or Mendelian randomization (MR) using quantitative trait loci as instrumental variables, then an antagonist of said gene may be a repurposing opportunity. Directional anchor genes then act as seed genes to define a network of other genes that interact with them. SNPs mapped to this network are then utilized to construct a pharmagenic enrichment score (PES) for the network. In the case of a binary disease phenotype, the interpretation of the PES would be that individuals with an elevated score relative to an appropriate population reference may benefit from a compound which modulates the directional anchor gene. The hypothesis underlying this is that these individuals will have genetic risk that impacts upstream or downstream processes relating to the directional anchor, as well as the anchor gene itself, which could be addressed by the repurposing candidate in question.

covariance between mRNA or protein expression of the gene and the GWAS trait of interest. We utilized a conservative method for multiple-testing correction whereby the Bonferroni methodology was implemented to divide the alpha level (0.05) by the total number of significantly *cis*-heritable models of genetically regulated expression (GReX) tested from any brain tissue considered or whole blood (supplemental material and methods, Tables S1–S4). Several genes had GReX available in multiple tissues, thus rendering Bonferroni correction conservative; however, we implemented this approach to capture only the most confidently associated genes that could constitute drug-repurposing candidates. We acknowledge that less conservative multiple-testing correction methods could be employed to account for the correlation observed between GReX models, such as a permutation-based approach.[26] For candidate directional anchor genes derived from TWAS/PWAS, we probabilistically finemapped those regions using the FOCUS methodology using the default prior ($p = 1 \times 10^{-3}$) and prior variance ($n\sigma^2 = 40$) to approximate Bayes' factors, such that the posterior inclusion probability (PIP) of each gene being a member of a credible set with 90% probability of containing the causal gene could be derived.[27] Genes with at least moderate finemapping support ($PIP > 0.4$) were taken forward as candidate directional anchor genes. We also investigated the impact of using a more conservative prior as outlined in the supplemental material and methods. Moreover, we tested whether SNPs that constitute the GReX model and either SZ or BIP displayed statistical colocalization with the *coloc* package as implemented by FUSION.[28]

Mendelian randomization

In addition, we leveraged variants strongly correlated with mRNA (expression quantitative trait loci [eQTL]) and protein expression (protein expression quantitative trait loci [pQTL]), respectively, as instrumental variables (IVs) in a two-sample Mendelian randomization (MR) analysis.[29] The MR approach was deployed to try and identify additional candidate directional anchor genes, as well as provide additional validation to TWAS/PWAS identified genes. Analogous to the TWAS/PWAS, eQTL/pQTL were derived from brain (MetaBrain, ROSMAP) and blood (eQTLGen, Zheng et al.[29]), with full details described in the supplemental material and methods.[29–32] Strict selection criteria were implemented to select suitable IVs, including retaining only independent genome-wide significant ($p < 5 \times 10^{-8}$) SNPs that were associated with three or fewer mRNA/proteins in each relevant tissue/study (supplemental material and methods). Moreover, we utilized a more stringent LD clumping procedure for eQTLs, given that the greater power and sample sizes for these datasets also results in immense pleiotropy among the SNP effects on mRNA. This was achieved by selecting only the most significant independent

SNPs using one megabase clumps, with LD estimated using the 1000 Genomes phase 3 panel. The effect of mRNA or protein expression for any given gene on SZ or BIP was estimated using the Wald ratio (single IV) or an inverse-variance weighted estimator (multiple IVs, with fixed effects due to the small number of IVs). As in the TWAS/PWAS, we utilized Bonferroni correction across all tissues in the mRNA and protein analyses, respectively, and then sought to identify candidate directional anchor genes from these signals. For any candidate directional anchor genes, where an approved drug was predicted to reverse the odds increasing direction of expression, we performed a series of sensitivity analyses in order to refine which genes would be suitable candidate directional anchors, as described more extensively in the supplemental material and methods. In brief, these involved assessing the genomic locus of the IV SNP, for genes it may be associated with, by testing for evidence of a shared causal variant through colocalization (default priors) and conducting a phenome-wide Mendelian randomization analysis (MR-pheWAS) using SNP effects from each trait in the IEUGWAS database. The above MR and sensitivity analyses were performed using the R packages TwoSampleMR v.0.5.5, ieugwasr v.0.1.5, and coloc v.4.0.4.[28,33]

Identifying drug-repurposing candidates

We searched genes prioritized from the TWAS/PWAS or MR analyses in the Drug-gene interaction database (DGIdb v.4.2.0, accessed April 2021) to identify approved compounds that could reverse the odds increasing direction of expression for SZ or BIP.[34] DGIdb combines data from databases such as DrugBank as well as curated literature sources. We defined high-confidence drug-gene interactions as those reported in DrugBank as well as at least one other database or literature source.

Identification of genes interacting with candidate directional anchor genes

Protein-protein interaction data were downloaded from the STRING database v.11.[35] We utilized each of the six candidate directional anchor genes as a seed gene, separately, and constructed a network of genes predicted to interact with the seed gene by retaining high confidence edges (confidence score >0.7) derived from experimental evidence or curated protein-complex and pathway databases, as this is generally considered the most rigorous evidence from STRING. Highly expressed nodes from systems throughout the body were retained to capture the impact of biological processes the brain and other organs. We then tested which gene-sets curated by the g:Profiler (v. e104_eg51_p15_3922dba) resource (GO, KEGG, Reactome, WikiPathways, TRANSFAC, miRTarBase, Human Protein Atlas, CORUM, and Human phenotype ontology) were overrepresented among the genes in each network, using the g:SCS (set counts and sizes) multiple-testing correction method implemented by g:Profiler that has been shown to better account for the complex, overlapping nature of these data.[36] We considered a corrected p value <0.05 as significant.

We then tested the association of the genes in each of these networks, with and without the directional anchor gene removed, with the common variant signal in the SZ and BIP GWAS using MAGMA v.1.09.[37] In brief, SNP-wise p values were aggregated at gene level, with SNPs annotated to genes using two different sets of genic boundary extensions to capture potential regulatory variation, conservative (5 kilobases [kb] upstream, 1.5 kb downstream), and liberal (35 kb upstream, 10 kb downstream). Gene-set association is implemented by MAGMA using linear regression, whereby the probit transformed genic p values (Z scores) are the outcome with a binary explanatory variable indicating whether a gene is in the set to be tested (β_S), covaried for other confounders like gene size, as described previously. The test statistic of interest is a one-sided test of whether $\beta_S > 0$, and thus quantifies whether the genes in the set are more associated than all other genes. We also investigated the association of the approximately 34,000 gene-sets collated by g:Profiler, such that we could demonstrate whether gene-sets overrepresented in each network were also associated with SZ or BIP.

Constructing directional anchor gene network PES

We sought to utilize variants annotated to the genes within the network of each candidate directional anchor genes to develop pharmagenic enrichment scores for SZ and BIP. As described previously, a PES is analogous to a genome-wide PRS in its derivation, with the key defense that it only utilizes variants mapped to the gene set of interest (Equation 1).[18] Specifically, a PES profile in individual i comprises the sum of the effect size of j variants from the GWAS ($\hat{\beta}_j$) annotated to at least one gene in set M, multiplied by the allelic dosage under an additive model ($G_{ij} \mid G = 0, 1, 2$).

$$PES_i = \sum_{j=1}^{M} \hat{\beta}_j G_{ij} \quad \text{(Equation 1)}$$

The genome-wide PRSs for SZ and BIP are essentially the same model but M incorporates the entire genome. In accordance with the MAGMA analyses, we tested two genic boundary configurations for evaluating the best-performing PES for each directional anchor gene network—conservative and liberal. Our previous PES-related approaches utilized the LD clumping and thresholding (C+T) approach, whereby SNPs are "clumped" such that the retained SNPs are largely independent and "thresholded" based on their association p value in the GWAS. In each case the threshold was set at the optimum for the druggable gene-set association at the population level. However, given that we selected the gene sets in this study based on interactions with the candidate directional anchor gene, we tested four different p value thresholds ($P_T \mid T = \{0.005, 0.05, 0.5, 1\}$), which represent a model with all SNPs, nominally significant SNPs, and a threshold an order of magnitude above or below the nominal threshold. These choices of T have been discussed extensively elsewhere.[18,19,38] We utilized PRSice-2 v.2.3.3 (linux) for the C+T models.[39] In addition, we utilized a penalized regression framework to shrink SNP effect sizes to optimize the model for each PES, as implemented by the standalone version of lassosum v.0.4.5.[40] The implementation for this method has been outlined extensively elsewhere, with the optimal tuning parameter (λ) based on the score that displays the highest correlation with the phenotype and the best performing constraint parameter (s) chosen from a range of *a priori* specified values to decrease computational burden (0.2, 0.5, 0.9, and 1).

Training and validation of directional anchor gene network PES

We utilized the prospective UK Biobank (UKBB) cohort (project ID = 58432) to define the best performing PES for each directional anchor gene network (approved by the UKBB access committee). Our group has previously processed the UKBB genotype data such that unrelated individuals of white British ancestry were

retained, along with other sample- and variant-level quality control considerations applied.[38] As a result, the composition of the full UKBB cohort in this study was 336,896 participants for which up to 13,568,914 autosomal variants were available (imputation INFO >0.8). SZ- and BIP-affected individuals were defined in the UKBB using a combination of self-report data both from the general assessment visit and the mental health questionnaire (MHQ), along with hospital inpatient data (primary or secondary ICD-10 codes), with full details in the supplemental material and methods. In total, there were 631 UKBB participants from the study cohort defined as having SZ, with 1,657 BIP-affected individuals identified. The control subjects were double the number of the respective case cohorts randomly, and independently for SZ and BIP, derived from 75,201 individuals with genotype data that completed the MHQ and did not self-report any mental illness. The full complement of SZ-affected individuals with the aforementioned controls ($n = 1,262$) was utilized as the training set for the SZ scores given the relatively small number of affected individuals. As a result, we utilized the Australian Schizophrenia Research Bank (ASRB) cohort as a validation set to attempt to replicate the associations observed with the scores, which has been described elsewhere.[18,41] The ASRB was a component of the PGC3 SZ GWAS, and so we retrained all the best performing PES scores using summary statistics with the ASRB cohort removed before testing them in that dataset. The BIP analyses employed a 70/30 split for the training and validation cohort in the UKBB, with double the number of independent MHQ-derived healthy control subjects utilized for each case-set. Further information regarding the demographic composition of these cohorts is provided in the supplemental material and methods.

The PES and PRS constructed using the C+T configurations and penalized regression were scaled to have a mean of zero and unit variance before evaluating their association with SZ or BIP, for the respective scores in the UKBB training cohorts, using binomial logistic regression covaried for sex, age, genotyping batch, and five SNP-derived principal components. The optimal PES for each network was selected for each disorder separately by calculating the variance explained on the liability scale (Nagelkerke's R^2, converted to the liability scale), assuming a 0.7% and 1% prevalence for SZ and BIP, respectively.[42] These PESs/PRSs that explained the most phenotypic variance were then profiled and tested in the validation sets. For PESs that were significantly associated with either disorder, we conservatively constructed another model that also included genome-wide PRSs, with a χ^2 test of residual deviance performed to ascertain whether adding the PES in addition to the PRS significantly improved model fit. Correlations (Pearson's) among scaled PESs and PRSs were visualized using the corrplot package v.0.84. Individuals with at least one elevated PES in the training cohorts (highest decile) were identified, with this binary variable tested for association with SZ or BIP using another logistic regression model. Finally, we also considered residualized PESs, whereby the residuals were extracted and scaled from a linear model that regressed genome-wide PRSs against principal components and genotyping batch on the score in question. All analyses described in this paragraph were performed utilizing R v.3.6.0.

Biochemical and mental health phenome-wide association studies

We then investigated the correlations between the best performing PES for each network and (1) blood or urine biochemical traits and (2) self-reported mental health disorders besides SZ or BIP. The biochemical analyses were performed in up to 70,625 individuals who did not self-report any mental health disorders in the MHQ and were also not included in the SZ or BIP training/validation sets as control subjects. There were 33 biochemical traits tested (raw values in Table S5) in a linear model with each PES or PRS as an explanatory variable covaried for sex, age, sex × age, age^2, 10 principal components, and genotyping batch. We also performed sex-stratified analyses, with oestradiol additionally considered in females. A number of sensitivity analyses were performed for PES-biochemical trait pairs that were significantly correlated after FDR correction: (1) adjustment for genome-wide PRS, (2) natural log transformation of the biochemical outcome variable, (3) inverse-rank normal transformed residuals as the outcome variable from a model that regressed sex, sex × age, and age^2, and (4) adjustment for statin use (given the number of lipid related signals uncovered). These correlations are observational in nature, and thus, there are several other potential confounders that could be considered; however, given the potential biases induced by adjusting for heritable covariates, we utilized the above strategies as a baseline suite of sensitivity analyses.[43] A specific test of sexual dimorphisms between the regression results in males and females was also performed based on the sex-specific regression estimates and standard errors, as outlined elsewhere.[44] Moreover, we then evaluated the association between each score and 14 non-SZ or BIP mental health disorders which individuals who completed the MHQ were asked to self-report (Table S6). In all instances, we used the 70,625 individuals who did not self-report any mental disorders as the control subjects in binomial logistic regression models covaried for the same terms as in the biochemical analyses.

Results

Candidate directional anchor genes reveal drug-repurposing opportunities in psychiatry

We sought to identify candidate directional anchor genes for SZ or BIP by integrating GWAS summary statistics for these traits with transcriptomic and proteomic data collected from either blood or post-mortem brain (Figure 2A). Specifically, we utilized genetically imputed models of mRNA or protein expression to conduct a TWAS and PWAS, respectively (Tables S1–S4). Genome-wide significant eQTLs and pQTLs were also leveraged as instrumental variables in a two-sample Mendelian randomization analysis to explicitly test for any causal effects of mRNA or protein expression, which is a more conservative paradigm (Tables S7–S10). After implementing Bonferroni correction within each analysis set (TWAS, PWAS, eQTL-MR, pQTL-MR), we found several genes for which their expression was associated with at least one of the psychiatric phenotypes at the mRNA or protein level that was also putatively modulated by an approved compound in a risk decreasing direction. First, we considered the TWAS results. There were 13 druggable genes from TWASs for which the direction of genetically predicted mRNA expression correlated with SZ could be pharmacologically counteracted, while there were two such genes for BIP, some examples of which are visualized in Figure 2B. For instance, imputed mRNA expression of the

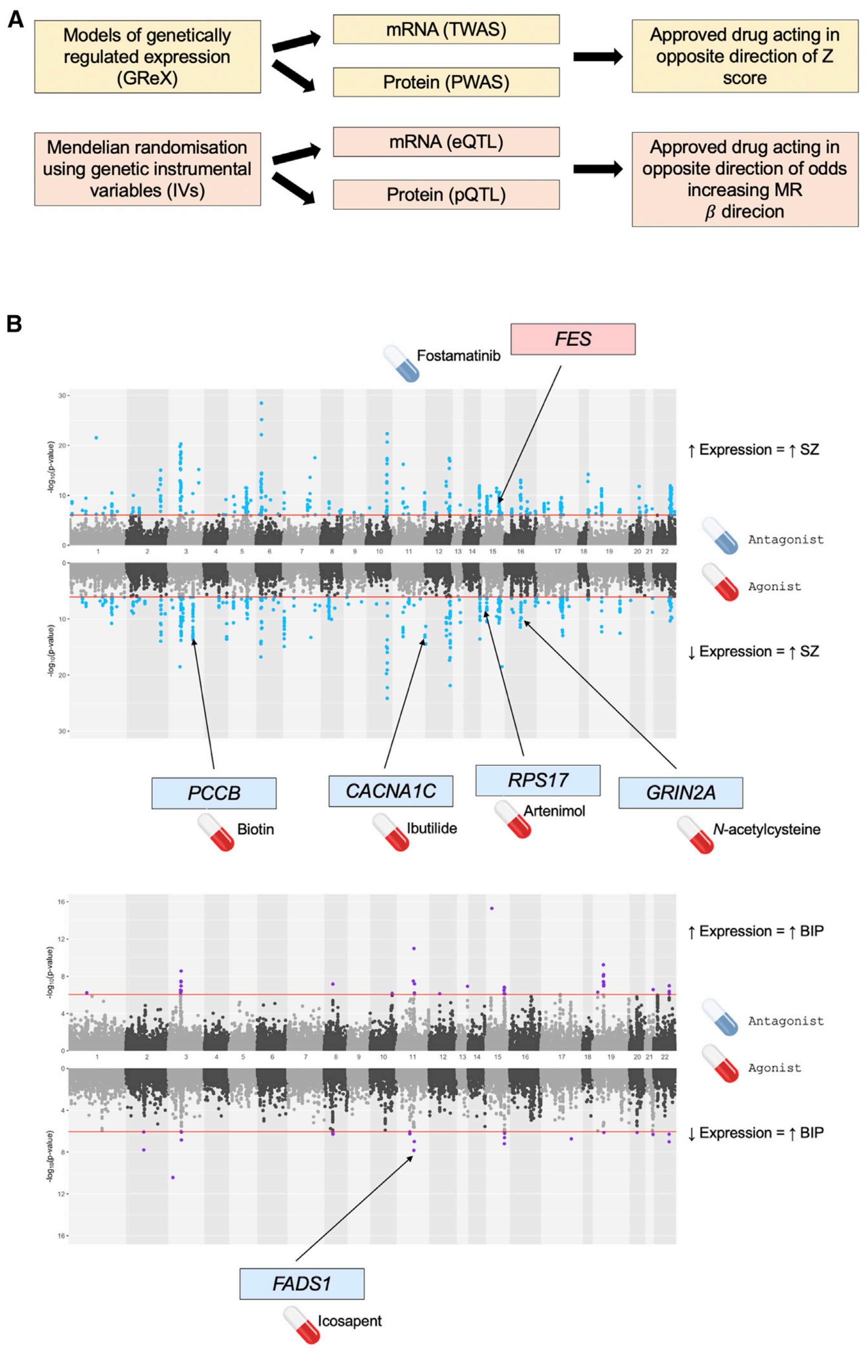

Figure 2. Identification of candidate directional anchor genes for schizophrenia and bipolar through integration of transcriptomic and proteomic data
(A) Schematic for the prioritization of candidate directional anchor genes through models of genetically regulated expression (GReX, yellow) and Mendelian randomization (orange). In both instances, approved compounds are derived for implicated genes that reverse the odds increasing direction of mRNA or protein expression. TWAS, transcriptome-wide association study; PWAS, proteome-wide association study.
(B) Results of the multi-tissue (brain and blood) TWAS for schizophrenia (SZ, top) and bipolar disorder (BIP, bottom). The Miami plot visualizes the −log10 transformed p value of association with genes exhibiting a negative genetic covariance between expression and the trait, that is, TWAS $Z < 0$, plotted in the downward direction. The red line denotes the Bonferroni threshold. The candidate directional anchor genes from the TWAS approach are highlighted on the plot along with their putative repurposing candidate that corrects the odds-increasing direction of expression. For example, predicted *PCCB* expression is negatively correlated with SZ, and thus, a *PCCB* agonist like biotin may be clinically useful.

calcium voltage-gated channel subunit gene *CACNA1C* (MIM: 114205) was negatively correlated with SZ ($p = 3.65 \times 10^{-15}$), and thus, an activator of this gene (like the antiarrhythmic agent Ibutilide) may be a repurposing candidate for SZ; this gene also has statistically significant association with BIP in the concordant direction but did not survive multiple-testing correction ($p = 3.18 \times 10^{-5}$). We compared the TWAS results to that of a PWAS using data from blood or brain tissue, although the number of proteins assayed in these studies was considerably smaller than that of the number of mRNA available, and thus, most of the candidate genes derived using the TWAS did not have protein measurements available for a direct comparison of the effect of protein expression relative to mRNA. However, there were two Bonferroni-significant TWAS genes that represented a plausible repurposing candidate with protein expression data available (*NEK4* [MIM: 601959] and *CTSS* [MIM: 116845]), with both genes showing a similar strength of association in the PWAS. For any given genetically predicted mRNA or protein expression, the gene is not necessarily causal due to LD complexity and other phenomena such as co-regulation.[45] This is an important consideration when using these approaches to direct drug repurposing, as the target the genes need to correspond to the genetic association for the disorder. As a result, we implemented a Bayesian fine-mapping procedure for each TWAS candidate gene to identify plausible causal genes in each locus (supplemental material and methods). We found four repurposing candidate genes for SZ with strong evidence for membership of a credible set with 90% probability of containing the causal gene (*PIP* > 0.8 – *PCCB* [MIM: 232050], *GRIN2A* [MIM: 138253], *FES* [MIM: 190030], and *CACNA1D* [MIM: 114206]). However, *CACNA1D* was excluded from further analyses due to the poor performance of its imputed model and complexity of its locus on chromosome three, as

Table 1. Candidate directional anchor genes for schizophrenia and bipolar along with their associated drug-repurposing candidates

Gene	Disorder	Protective direction	Repurposing candidates	Sensitivity analyses
PCCB	SZ	increased expression	biotin	high-confidence causal gene from TWAS ($PIP > 0.8$), further supported by MR
FADS1	BIP	increased expression	icosapent, linolenic acid	moderate-confidence causal gene from TWAS ($PIP > 0.4$, additionally supported by colocalization – $PP_{H4} > 0.8$), further supported by MR
GRIN2A	SZ	increased expression	*N*-acetylcysteine, glycine	high-confidence causal gene from TWAS ($PIP > 0.8$)
CACNA1C	SZ	increased expression	ibutilide	moderate-confidence causal gene from TWAS ($PIP > 0.4$, additionally supported by colocalization – $PP_{H4} > 0.8$)
RPS17	SZ	increased expression	artenimol	moderate-confidence causal gene from TWAS ($PIP > 0.4$, additionally supported by colocalization – $PP_{H4} > 0.8$)
FES	SZ	decreased expression	Fostamatinib, Lorlatinib	high-confidence causal gene from TWAS ($PIP > 0.8$)

outlined more extensively in the supplemental material and methods. We then considered a more lenient posterior inclusion probability of 0.4, which identified two more genes for SZ (*CACNA1C* and *RPS17* [MIM: 180472]) and one BIP gene (*FADS1* [MIM: 606148]). Colocalization analyses were also performed to test a related but distinct hypothesis that the GWAS signal and SNP weights in the expression model share an underlying single causal variant. Interestingly, for the genes selected using the lower confidence $PIP > 0.4$ threshold, we found strong evidence for a shared causal variant ($PP_{H4} > 0.9$), supporting their inclusion as putative drug-repurposing targets. Specifically, there was particularly strong evidence that the *cis*-acting SNPs used to construct the *CACNA1C* model shared a causal variant with schizophrenia in the cerebellum ($PP_{H4} = 0.946$), with slightly weaker support for this also in the substantia nigra ($PP_{H4} = 0.777$). *RPS17* also demonstrated strong evidence for colocalization between the TWAS weights and schizophrenia in the PsychENCODE cortical dataset ($PP_{H4} = 0.953$), which was also seen for *FADS1* and bipolar disorder ($PP_{H4} = 0.95$). We did not consider the two genes shared with the PWAS any further as they did not display strong finemapping support in the TWAS, which is a more accurate representation of any given locus due to the more expansive number of genes with RNA-seq available. In summary, using a genetically imputed expression approach (TWAS/PWAS), we identified five candidate directional anchor genes for SZ and one for BIP (Table 1). For example, imputed *GRIN2A* mRNA expression was negatively correlated with SZ ($p = 1.44 \times 10^{-9}$), with a trend also observed for BIP ($p = 5.07 \times 10^{-3}$), with compounds of interest in psychiatry, such as *N*-acetylcysteine, known to agonize this subunit.[46,47]

To attempt to prioritize additional candidate directional anchor genes, as well as support the TWAS/PWAS results, we then utilized eQTL and pQTL as IVs to estimate the causal effect of mRNA or protein expression on either disorder outcome in a Mendelian randomization analysis, given more onerous assumptions are met (supplemental material and methods, Tables S7–S10). Conservatism in this context is critical, as the use of molecular QTLs related to variables like mRNA expression as IVs is challenging due to LD complexity and the potential effect of QTLs on multiple genes.[29,48] As a result, we implemented conservative selection criteria for an eQTL or pQTL to be an IV, particularly in the case of eQTLs where sample sizes for some tissues are now very large. Independent SNPs (LD $r^2 < 0.001$) acting as eQTLs or pQTLs at a threshold of genome-wide significance ($p < 5 \times 10^{-8}$) were selected from post-mortem brain or blood, as outlined in the material and methods and supplementary materials. Due to the conservative nature of these analyses, many of the genes considered in the TWAS/PWAS did not have a suitable IV available. Conversely, a small number of genes that did not display adequate multivariate *cis*-heritability in the TWAS/PWAS weights could now be included. The mRNA models after Bonferroni correction uncovered four genes for which expression exerted a potential causal effect on SZ or BIP with a suitable compound approved to reverse the odds-increasing direction of effect. There were three for SZ (*PCCB*, *NEK1* [MIM: 604588], and *PTK2B* [MIM: 601212]), as well as *FADS1* for bipolar. Interestingly, *PCCB* and *FADS1* overlapped with the TWAS results—as an example, each standard deviation increase in cortical *FADS1* expression was associated with approximately 15.23% (95% CI: 8.69%, 21.77%) decrease in the odds of BIP, which could be accentuated by a *FADS1* agonist like the omega-3 fatty acid supplement icosapent (Ethyl eicosapentaenoic acid). We then performed a series of sensitivity analyses to assess IV validity and for evidence of confounding pleiotropy (supplemental material and methods). These analyses supported *PCCB* and *FADS1* as candidate directional anchor genes but did not support assigning *NEK1* or *PTK2B* as a directional anchor gene, as outlined in the supplemental material and methods. The index IV-SNPs mapped to *PCCB* and *FADS1* expression, respectively, was then utilized to perform a phenome-wide scan spanning over 10,000 GWAS of the effect of expression of these two genes using SNP effect sizes from the IEUGWAS

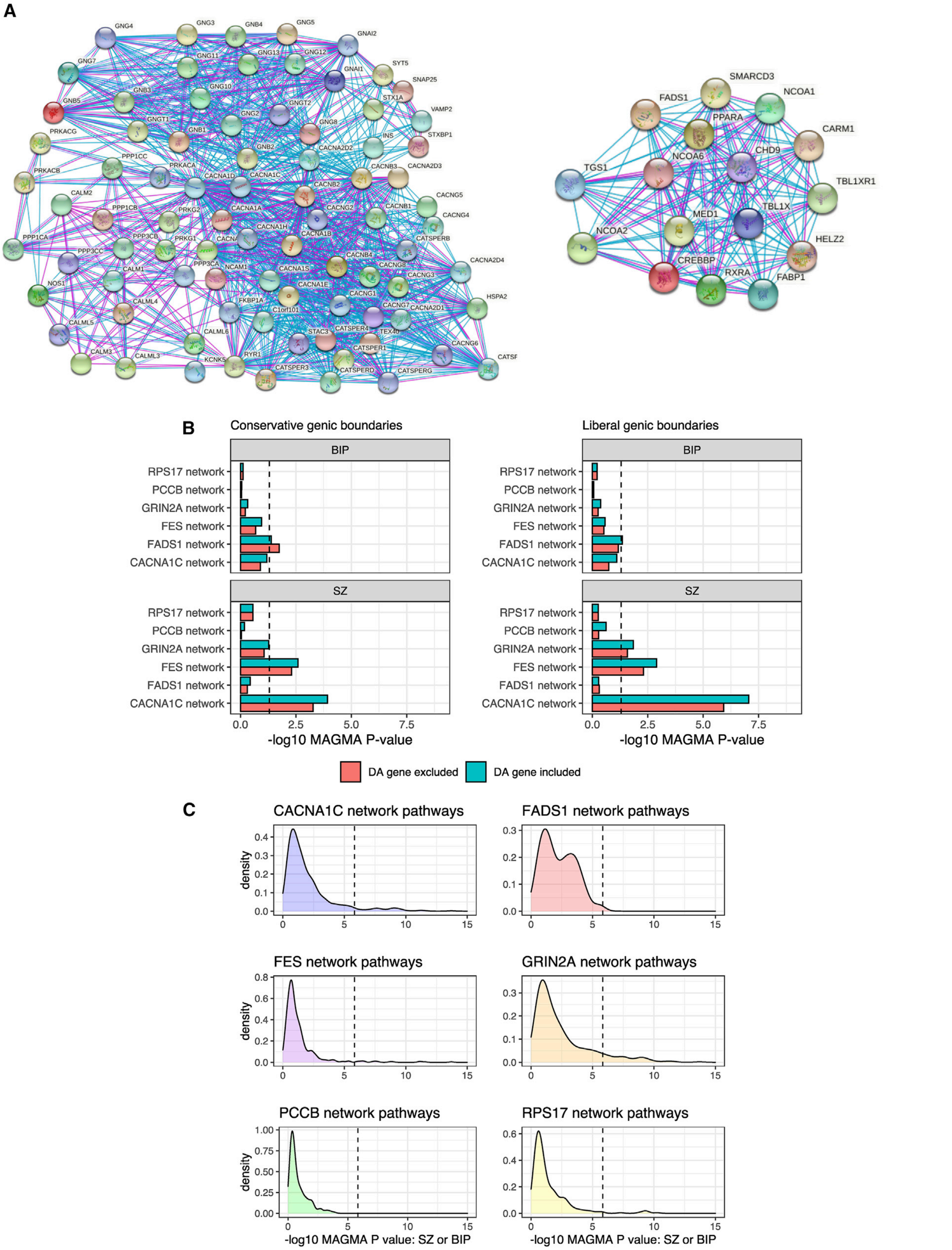

Figure 3. Biological networks interacting with candidate directional anchor genes
(A) Visualization of two networks of genes that putatively interact with *CACNA1C* (left) and *FADS1* (right) based on experimental and curated database evidence. Blue edges represent evidence from curated databases, while purple edges denote experimentally determined evidence.

(legend continued on next page)

database (Tables S11–S13). First, we found that increased cortical expression of *PCCB*, which was associated with deceased odds of SZ from a previous GWAS, was also linked to a reduction in other psychiatric phenotypes from self-reported UK Biobank GWAS such as worry, neuroticism, nervousness, and tenseness, supporting the utility of a *PCCB* agonist, like biotin, as a repurposing candidate. Second, the phenome-wide data for increased cortical *FADS1* expression demonstrated, as expected, a strong effect on lipids, including increased HDL and decreased triglycerides. Considering the pQTL results, another potential candidate for BIP (MAP2K2) was suggested using a *trans*-pQTL as IV, but it was excluded as a directional anchor gene to retain the most biologically confident associations, as *trans* acting signals are more difficult to interpret. Although the MR approach did not add any additional candidate directional anchor genes (after exclusion of MAP2K2), it provided more support to *PCCB* and *FADS1*. A less conservative MR paradigm in terms of IV selection would likely yield more genes but as our TWAS/PWAS analyses were already discovery focused, we believe this would not be appropriate given the underlying assumptions of MR. We summarize the candidate directional anchor genes in Table 1.

We also considered two factors that are directly relevant to the clinical relevance of these prioritized compounds for schizophrenia and bipolar disorder that target a directional anchor: ability to cross the blood-brain barrier (BBB) and severity of side effects. First, using a curated database of BBB permeability,[49] biotin, glycine, and ibutilide were predicted to cross the BBB. *N*-acetylcysteine was classified as not BBB permeable in the database; however, evidence from the literature demonstrates that an amide derivative of *N*-acetylcysteine is BBB permeable.[50] The fatty acid compounds and lorlatinib were not in the database but are known to cross the BBB, particularly lorlatinib, as it was designed explicitly to be BBB permeable.[51,52] The other compound predicted to target *FES* in addition to lorlatinib, fostamatinib, was not predicted to cross the BBB in the database. Finally, artenimol was also not in the database but is reported in the literature as BBB permeable in rodent models.[53] We then considered the adverse effects for each compound recorded in SIDER, DrugBank, or the wider literature.[54,55] Biotin is one of the B vitamins and has a safe profile. Like any vitamin, toxicity at high doses can occur, but its water-soluble nature means that excess concentrations are usually efficiently excreted through urine. The fatty acids that target *FADS1* also have a safe profile, with icosapent reported in clinical trials to not show an elevation of treatment-emergent adverse effects in intervention arms versus placebo.[56] *N*-acetylcysteine presents also with relatively mild adverse effects, including flushing and hives in approximately 6% of individuals, although previous clinical trials of this compound as an adjuvant in bipolar disorder have reported an elevated incidence of mild gastrointestinal symptoms like heartburn and vomiting.[57] The *CACNA1C* agonist ibutilide unsurprisingly is associated with cardiac adverse effects like ventricular extrasystoles in approximately 5% of individuals, as well as tachycardia. Artenimol is also relatively safe, although linked to more serious hepatic and cardiac pathologies in rare cases. Finally, the two compounds that target *FES* are antineoplastic drugs, and thus, can have severe side effects at therapeutic doses for cancer. However, chemotherapeutic drugs, like the retinoid analog bexarotene, have previously shown promise in clinical trials for psychiatric illness at much lower doses than used in oncology.[17,58]

Interaction networks related to directional anchor genes capture biology associated with schizophrenia and bipolar disorder

We sought to define a network of genes that display high-confidence interactions with each candidate directional anchor gene using data from the STRING database, such that we can then construct a pharmagenic enrichment score using variants annotated to these genes. The number of direct interactions identified for each of the six candidate genes, excluding the gene itself, were as follows (Table S14): *CACNA1C* network (83 genes), *FADS1* network (16 genes), *FES* network (37 genes), *GRIN2A* network (54 genes), *PCCB* network (26 genes), and *RPS17* network (254 genes). The genes in each of these networks displayed significantly more interactions within the respective networks than what would be expected by chance alone for a set of randomly drawn proteins ($p < 1 \times 10^{-16}$, Table S15), with an example of two of these networks (*CACNA1C* and *FADS1*) visualized in Figure 3A. These networks likely represent heterogenous biological processes in which the directional anchor gene may participate, and thus, we sought to better understand the biology of these interacting genes by testing their overrepresentation within biological pathways and other ontological gene sets. The six directional anchor gene networks each displayed overrepresentation in pathways related to the known biology of the candidate gene (Tables S16–S21). For instance, the *CACNA1C* network genes were enriched

(B) Gene-set association (MAGMA) of the entire network for each candidate directional anchor (DA) gene, with and without the DA gene included from the model. Dotted line represents nominal significance ($p < 0.05$). The MAGMA p value is derived from a model that tests whether the common variant signal within genes in the network is greater than what is observed among all remaining genes. Two genic boundaries were utilized to annotate SNPs to genes from the GWAS: conservative (5 kb upstream, 1.5 kb downstream, left panel) and liberal (35 kb upstream, 10 kb downstream, right panel).
(C) Kernel density estimation plots of the MAGMA gene-set association p value for each gene set tested using either schizophrenia or bipolar results, whichever was more significant, which had a significant overrepresentation of genes within that network. The dotted line represents the Bonferroni significance level for approximately 34,000 gene sets considered in the full analysis of all gene sets that were tested for overrepresentation.

within several hundred gene sets, many of which related to neuronal calcium channel biology along with systemic processes known to involve calcium signaling such as pancreatic insulin secretion. Furthermore, the *FADS1* network genes displayed an overrepresentation in several lipid- and other metabolic-related pathways, while *GRIN2A* network genes demonstrated a strong link to neuronal biology.

We then sought to test two specific characteristics regarding the relationship of these networks to SZ and BIP at the population level: first, whether these networks were enriched with the common variant signal for either disorder, even after removing the directional anchor, and second, whether specific biological pathways captured by these networks were associated with SZ or BIP. We first examined the overall network enrichment using MAGMA, which is a test of competitive association in the network verses all other genes (Figure 3B, Table S22). The *CACNA1C* network was strongly associated with SZ ($p = 8.87 \times 10^{-8}$), even after removing *CACNA1C* itself ($p = 1.19 \times 10^{-6}$). The *FES* and *GRIN2A* networks demonstrated a nominal enrichment of the SZ common variant signal relative to all other genes, $p = 1.28 \times 10^{-3}$ and $p = 0.014$, respectively, remaining significant upon removing the relevant directional anchor genes. None of the other networks were associated with SZ when considering all genes, with only the *FADS1* network demonstrating a nominal association with BIP ($p = 0.04$). Given that these networks represent several different biological processes, we further hypothesized that specific gene sets for which they were overrepresented may specifically display a stronger association with SZ or BIP, which relates to the second characteristic described above (Tables S23–S28). Indeed, we show that all of the networks had at least one overrepresented pathway that was associated with SZ or BIP using Bonferroni (FWER < 0.05) and Benjamini-Hochberg (FDR < 0.05) correction, with the exception of the sets enriched in the *PCCB* network that only survived correction using FDR. Kernel density estimation plots of the MAGMA gene-set association p values are visualized in Figure 3C, which show pathway-associations reaching these thresholds. We briefly describe the results for the *CACNA1C* and *GRIN2A* networks below for illustration. Pathways overrepresented in the *CACNA1C* network related to calcium channel activity displayed strong association with SZ, for instance, voltage gated calcium channel process ($p = 2.80 \times 10^{-10}$, $q = 6.83 \times 10^{-7}$), while the regulation insulin secretion pathway that also was enriched in the network was associated with SZ and trended towards surviving multiple testing correction for BIP. *GRIN2A* network members also displayed an enrichment among several neuronal pathways strongly associated with SZ, such as synaptic signaling ($p = 3.88 \times 10^{-8}$, $q = 2.82 \times 10^{-5}$). Taken together, these results suggest that pathways in which genes in each network participate are associated with psychiatric illness and reinforces the biological salience of these networks.

Directional anchor gene network pharmagenic enrichment scores display significant trait associations after adjustment for genome-wide polygenic risk score

Pharmagenic enrichment scores (PESs) were then constructed for the genes in each directional anchor gene network using SNP weights for SZ and BIP, respectively. SZ and BIP PESs were considered for all six networks given the high genetic correlation between SZ and BIP, as well as extensive phenotypic overlap. We defined a training set of individuals affected with SZ ($n = 631$) and BIP ($n = 1{,}161$) in the UK Biobank, with double the number of control subjects randomly, and independently, selected from individuals with no self-reported mental health conditions for each training set. Two methods were utilized to find the most parsimonious PES profile for each network, along with a genome-wide PRS for SZ and BIP: clumping and thresholding (C+T) and penalized regression (Table 2).

Schizophrenia

In the SZ cohort, there were three network SZ PESs which were significantly associated with increased odds of SZ after multiple testing correction including networks for *FES*, *GRIN2A*, and *RPS17* (Table 2, Figure S1A). In the *GRIN2A* network, a PES featuring 5,037 variants constructed using penalized regression explained approximately 0.35% of phenotypic variance on the liability scale (OR per SD in score = 1.19 [95% CI: 1.09, 1.29], $p = 9.23 \times 10^{-4}$). We then conservatively adjusted for the best performing genome-wide SZ PRS and found that the *GRIN2A* network PES remained significantly associated with SZ. In the *FES* and *RPS17* networks, their respective PES were just below the threshold for significance after PRS adjustment (Table S29). It is notable that the SZ network PES profiles were only marginally correlated with genome-wide SZ PRS (all $r < 0.11$, Figure S2A), which suggests these scores may capture biologically aggregated risk which is distinct from the undifferentiated genome-wide signal. Interestingly, the majority of individuals with SZ (53.72%) had at least one elevated PES (for the disorder) in the highest decile, which was statistically significant even after covariation for genome-wide PRS – OR = 1.45 [95% CI: 1.22, 1.67], $p = 1.57 \times 10^{-3}$. Interestingly, among individuals in this cohort with relatively low SZ PRS (lowest decile), 12 out of the 19 SZ-affected individuals had an elevated PES (63%), with a nominally significant association remaining between elevated PES and SZ among those with low genome-wide PRS ($p = 0.027$). In other words, low genome-wide risk and high PES was associated with increased odds of schizophrenia relative to those with low genome-wide genetic risk without an elevated PES. Upon considering only SZ-affected individuals in terms of low PRS, we found that 46.88% had at least one elevated PES. Taken together, these data suggest that some individuals with otherwise low SZ PRS have localized genetic risk within these biological networks. Given the relatively small number of SZ-affected individuals in the UKBB, we sought to replicate our results using an independent case-control cohort from the ASRB ($n_{cases} = 425$, $n_{controls} = 251$)

Table 2. Characteristics of the best performing schizophrenia and bipolar genome-wide PRS along with a pharmagenic enrichment score for each directional anchor gene network

Score	n_{SNPs}[a]	Beta (SE)[b]	p	R^b	Type[c]
**Bipolar disorder**					
PRS	1,096,096	0.62 (0.04)	2.91×10^{-51}	4.96%	penalized regression
CACNA1C network	396	0.11 (0.04)	2.97×10^{-3}	0.16%	penalized regression
FADS1 network	196	0.06 (0.04)	0.1	0.05%	C+T
FES network	5	0.10 (0.04)	4.87×10^{-3}	0.14%	penalized regression
GRIN2A network	143	0.17 (0.04)	3.81×10^{-6}	0.39%	penalized regression
PCCB network	520	0.14 (0.04)	2.22×10^{-4}	0.25%	penalized regression
RPS17 network	13,724	0.15 (0.04)	1.07×10^{-4}	0.28%	penalized regression
**Schizophrenia**					
PRS	1,861,450	1.01 (0.07)	2.65×10^{-51}	9.55%	penalized regression
CACNA1C network	128	0.09 (0.05)	0.1	0.09%	C+T
FADS1 network	36	0.04 (0.05)	0.4	0.02%	C+T
FES network	141	0.17 (0.05)	1.41×10^{-3}	0.32%	C+T
GRIN2A network	5,037	0.18 (0.05)	9.23×10^{-4}	0.35%	penalized regression
PCCB network	2,393	0.07 (0.05)	0.16	0.06%	penalized regression
RPS17 network	77	0.15 (0.05)	3.53×10^{-3}	0.27%	penalized regression

[a]SNPs with a non-zero coefficient after reweighting in the penalized regression model or independent SNPs after linkage disequilibrium clumping and thresholding (C+T).
[b]Bipolar disorder or schizophrenia log odds per standard deviation increase in the score (standard error).
[c]The two models evaluated were clumping and thresholding (C+T) or penalized regression (as implemented by the lassosum package).

rather than splitting the UKBB cohort into a training and validation set. The PES and PRS models were retrained in the UKBB from the same GWAS with the ASRB cohort removed. We were able to nominally replicate the association of the *FES* network PES with SZ in the ASRB (OR per SD = 1.21 [95% CI: 1.04, 1.38], p = 0.024), while the observed association between the *GRIN2A* and *RPS17* network PES and SZ and in the UKBB was not replicated (Table S30).

Bipolar disorder

BIP PES within these networks was then profiled in the UKBB training set (Table 2, Figure S1B). Interestingly, there were more of the directional anchor gene network PES associated with BIP than SZ, which may have been a reflection of the greater statistical power afforded by the larger number of BIP-affected individuals in the UKBB. Specifically, all of the network BIP PESs were significantly higher in affected individuals, with the exception of the *FADS1* network PES for which there was only a trend towards significance. Analogous to the SZ cohort, the *GRIN2A* network PES explained the most phenotypic variance on the liability scale (0.39%), with each SD in the score associated with a 19% (95% CI: 12%, 26%) increase in the odds of BIP. Moreover, adjustment for BIP genome-wide PRS did not ablate the significance of the *GRIN2A* network, *RPS17* network PES, and *FES* network PES, while the *PCCB* network PES trended towards significance (p = 0.1) after PRS covariation (Table S31). The correlations between each PES and BIP PRS were also small (Figure S2B); however, the *RPS17* network PES ($r = 0.13$), *CACNA1C* network PES ($r = 0.14$), and *PCCB* network PES ($r = 0.13$) were slightly larger in terms of their PRS correlation than what was observed for the SZ scores. We then investigated the characteristics of individuals with elevated BIP PESs and found—like SZ—that almost half of the BIP-affected individuals (49.1%) had at least one PES greater than or equal to the 90th percentile. There was also enrichment of BIP-affected individuals among participants with an elevated PES after adjusting for BIP PRS (OR = 1.19 [95% CI: 1.04, 1.34], p = 0.027). Considering BIP-affected individuals in the lowest decile of the BIP PRS distribution, 36% of them had at least one top decile PES despite their low genome-wide burden, although unlike SZ the association between elevated PES and case-status in this subcohort was not statistically significant. An independent BIP case-control cohort from the UKBB was utilized to attempt to replicate these associations (Table S32), and we found that the network *RPS17* PES was significantly enriched in BIP-affected individuals in this validation cohort, while there was a trend for the *GRIN2A* network PES (p = 0.052).

Sensitivity analyses for the GRIN2A network PES

The *GRIN2A* network PES explained the most phenotypic variance for SZ and BIP and survived covariation for genome-wide PRS; therefore, we wanted to test whether

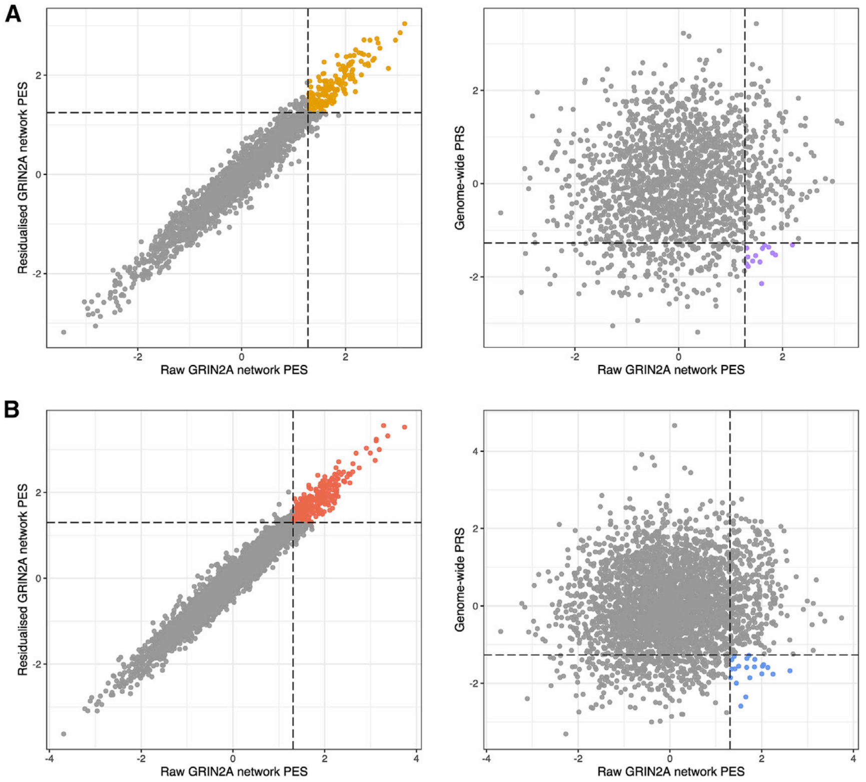

Figure 4. Schizophrenia and bipolar disorder *GRIN2A* directional anchor gene network pharmagenic enrichment scores and their relationship with PRS
The scatter plots denote the concordance between the scaled unadjusted (raw) *GRIN2A* network PES for SZ (A) and BIP (B) and both a residualized score and genome-wide PRS. Specifically, the left-most scatterplots visualize the relationship between the raw *GRIN2A* network PES and the residuals from a model that regressed genotyping batch, ten SNP-derived principal components, and genome-wide PRS for the disorder in question (residualized *GRIN2A* PES). The dotted lines represent the 90th percentile of the raw PES and residualized PES, respectively. The points colored orange (SZ) and red (BIP) indicate individuals with a PES in the 90th percentile or above for both the raw and residualized scores. The right scatterplots plot the relationship between genome-wide PRS for SZ or BIP and the *GRIN2A* network PESs. In these instances, the dotted vertical line denotes the 90th percentile of the *GRIN2A* PES, while the horizontal dotted line denotes the 10th percentile of genome-wide PRS. As a result, the points colored purple and blue in the SZ and BIP plots, respectively, are individuals with low relative genome-wide PRS (lowest decile) but high *GRIN2A* PES (highest decile).

constructing a PES for this network with *GRIN2A* excluded would still be associated. In other words, we investigated whether there was an effect from variants mapped to the network without the directional anchor gene itself. For example, the *GRIN2A* network PES with *GRIN2A* removed was still significantly enriched in both SZ and BIP ($p_{SZ} = 9.23 \times 10^{-4}$ and $p_{BIP} = 3.81 \times 10^{-6}$). The relationship between genome-wide PRS and this PES was also examined in further detail by constructing a "residualized PES" whereby we obtained the normalized residuals from a model that regressed SNP-derived principal components, genotyping batch, and genome-wide PRS for BIP and SZ, respectively, on the *GRIN2A* network PES for either disorder. We posit that the individuals with an elevated residualized PES are more likely to represent true enrichment in that network given that the effect of the genome-wide PRS, along with variables related to technical artefacts and population stratification, have been adjusted for. Encouragingly, we find that the correlation between the raw *GRIN2A* network PES for either disorder and their respective residualized PESs were highly concordant, with the majority of individuals with an elevated *GRIN2A* PES ($\geq$90th percentile) also in that same quantile for the residualized PES (Figures 4A and 4B).

Phenotypic correlations of directional anchor gene network scores support their biological relevance

We then investigated the association of the directional anchor gene PES in an independent subset of the UKBB with other mental health phenotypes and systemic biochemical measures (Tables S33–S35, Figure 5). The correlation profile of each PES relative to these phenotypes may also support its clinical utility, while it also provides an opportunity to further establish what distinct properties these scores have from a genome-wide PRS. First, all SZ and BIP network PESs, along with their respective PRS, were regressed against 33 blood and urine measures in up to 70,625 individuals, while estradiol in females was also additionally considered in a sex-stratified analysis (Figure 5A). In both sexes, we found that the *FADS1* network PES for SZ and BIP was significantly correlated with lipid-related traits after conservative Bonferroni correction for all PES/PES-trait pairs tested ($p < 1.11 \times 10^{-4}$). For instance, these *FADS1* network PESs were negatively correlated with HDL cholesterol and apolipoprotein A1 levels, whereas increase in the same PES was associated with higher measured triglycerides. The *FADS1* network PESs were also significantly associated other non-lipid biochemical traits including alkaline phosphatase, sex-hormone binding globulin (SHBG), and urate. Notably, adjusting the PES for a genome-wide PRS for SZ or BIP did not ablate its association with the trait, suggesting that these signals are not a product of genome-wide polygenic inflation (Tables S36–S39). To the contrary, there was evidence that PRS was correlated in the opposite direction with lipids to that of the corresponding PES. Given the strong lipid-related

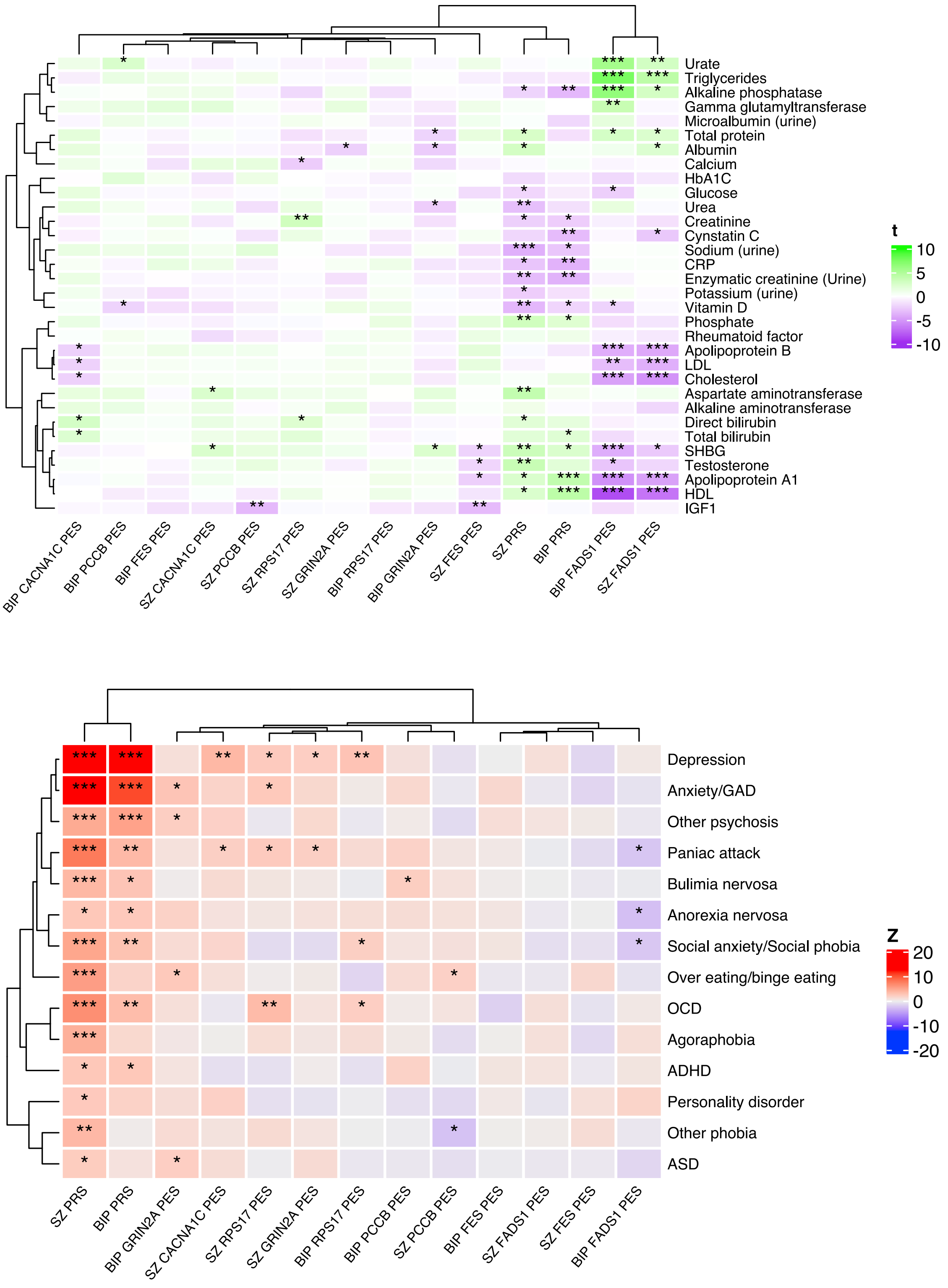

Figure 5. Phenome-wide association studies (pheWASs) of each network PES or PRS related to serum or urine biochemical measures and mental health disorders

Heatmap of the association between each network PES and PRS with each trait tested for the biochemical measures (top) and self-reported mental health disorders (bottom). Traits ordering derived from clustering by Pearson's distance. The variable visualized in the heatmaps for the continuous biochemical traits was the regression *t* value (beta/SE), while for the binary mental health phenotypes it was the corresponding *Z* value from the logistic regression, whereby $Z > 0$ equates to an odds ratio for the disorder >0. Asterisks were utilized to denote the significance of the association: *p < 0.05, **false discovery rate (FDR) > 0.05, and ***family-wise error rate (FWER) < 0.05.

signals, we also adjusted for statin use in an additional sensitivity analysis, but this similarly did not markedly impact the findings (Table S39). Using less stringent FDR correction (FDR < 0.05) revealed more PES association with biochemical measures. This included a negative correlation between both the *PCCB* and *FES* network PESs for SZ and insulin-like growth factor 1 (IGF1) as well as positive correlation between the *RPS17* network PES for SZ and creatinine. However, there was no direct effect of SZ or BIP PRS on IGF-1 or creatinine, with FES-related tyrosine kinase activity previously shown in the literature to be associated with IGF-1 biology.[59] Sex-stratified analyses identified even more PESs associated with a biochemical trait (Table S34)—for example, in males the SZ *PCCB* network PES was positively correlated with SHBG, which interestingly is in the opposite direction to the correlation of SHBG observed with the *FADS1* network PES, further highlighting biological heterogeneity among different networks. The BIP *CACNA1C* network PES in males was also positively correlated with direct bilirubin using an FDR cut-off, while the BIP *GRIN2A* network PES was negatively correlated with measured total protein. Finally, we formally tested for evidence of sexual dimorphic effects of PES/PRS on each biochemical measure and revealed nominal evidence of heterogeneity between sexes in these associations for some traits such as the effect of the *CACNA1C* network PES on direct bilirubin (Table S40).

We also performed a phenome-wide association study of each score with 14 self-reported mental health disorders in the UKBB cohort, excluding SZ and BIP (Figure 5, Table S35). The number of affected individuals ranged from 66 for attention deficit hyperactivity disorder (ADHD) to 22,974 for depression, with the same cohort of 70,625 individuals without a self-reported mental health condition not featured in the SZ or BIP training/validation sets leveraged as control subjects. Unsurprisingly, we found that SZ and BIP PRSs were strongly associated with increased odds of several mental health disorders after Bonferroni correction, but we also found network PESs associated with some of these phenotypes using FDR < 0.05 as the multiple-testing correction threshold. Specifically, there was an association between the SZ *CACNA1C* PES and increased odds of depression, while the SZ *RPS17* network PES was associated with increased odds of self-reported OCD. These disorders were also associated with elevated SZ PRS, but both PESs remained significantly higher in those with the respective self-reported phenotypes even after covariation for the effect of the SZ PRS. There were also several other nominal associations ($p < 0.05$), including one of particular interest in the case of the BIP *FADS1* network PES, for which a higher score displayed some evidence of a protective effect on self-reported anorexia nervosa. While this association does not survive multiple testing correction, and thus should be interpreted cautiously, it is notable as the *FADS1* network PES was associated with lipid profiles in an analogous direction to what has previously shown to be genetically correlated with anorexia nervosa GWASs via LD score regression.[11,21] In summary, these data coupled with the biochemical associations support the distinct nature of network PESs from PRSs and emphasize the unique insights that can be afforded by these partitioned scores.

Discussion

In this study, we leverage genetics to identify drug-repurposing candidates for psychotic disorders and show how they may be directed specifically to patients. This precision medicine approach is critical given that phenotypic and genetic heterogeneity confounds traditional interventions designed to target the entire disease population. We believe that a key advance in this study is that it provides a direct link between compounds with evidence for efficacy at the population level, a putative expression-related mechanism, and genetic risk scores in the network of genes that interact with the prioritized drug target.

Transcriptomic and proteomic data integrated with GWASs through GReX (TWAS/PWAS) and causal inference (MR) revealed six interesting target genes for SZ or BIP that could be modulated in a risk-decreasing direction by an approved drug. While we did not reveal pharmacological targets of existing therapies for either SZ or BIP, some of the implicated genes, including *GRIN2A* and *ANKK1* (MIM: 608774), had some weak evidence of being off-targets for antipsychotics after correction for multiple testing. Compounds that target *GRIN2A*, one of the genes prioritized for SZ, in the risk-decreasing direction have previously been subjected to randomized control trials as an adjuvant to antipsychotic treatment. Specifically, *N*-acetylcysteine and glycine intervention studies suggested that these compounds could be effective in improving multiple symptom domains including negative and cognitive-related dimensions of the disorder.[60,61] Omega-3 fatty acids, which are related to the biology of the BIP candidate gene *FADS1*, have also been of interest in that disorder, although trials have had contradictory results in terms of benefit.[62] This heterogeneity is unsurprising given the complexity of the BIP phenotype and demonstrates the utility of an approach such as ours that could more specifically target these interventions. The remaining genes and respective repurposing candidates all had plausible biological links to neuronal biology or psychiatric illness,[63–66] and thus warrant further investigation of their utility even without genetic stratification.

While these six candidates were supported by genetically regulated mRNA expression, this was not confirmed by genetically regulated protein expression and was probably due to reduced power to detect proteome-related associations, as these data are still relatively limited.[67,68] There are also some limitations to using both the GReX and MR frameworks for target identification,[7,45,48] although these are somewhat mitigated to a degree by the suite of sensitivity analyses we performed, including probabilistic

finemapping and colocalization, which strengthen our confidence in these six genes. Ideally, future statistical and molecular study of these association signals should be undertaken to refine our understanding of the role of these genes in the pathophysiology of SZ and BIP. Moreover, larger sample size panels of expression studies to estimate QTLs and GReX, as well as cell-type-specific data will also boost discovery power in these approaches.

We outlined a mechanism whereby genetic risk for the disorder could be profiled among the network of genes that were prioritized as repurposing opportunities and act as candidate directional anchor genes based on their direction of effect (network PES). Crucially, we observed a notable portion of cases with low overall PRS but at least one elevated PES, further supporting the biologically unique insights that may be gained from PES relative to an undifferentiated genome-wide approach. Most of the scores that we considered demonstrated at least nominal significance for association with either SZ or BIP, with PESs like the *GRIN2A* network PES significantly enriched in both SZ and BIP even after conservatively correcting for the effect of a genome-wide PRS. However, it should be noted that the training sets we used in this study were modestly powered, and larger training sets would be beneficial given that partitioned scores like a PES will have smaller overall effect sizes than a PRS. The relatively small trait-effect sizes of PESs in terms of their phenotypic variance explained does not also necessarily preclude individual level relevance, particularly because PESs like the *FADS1* network PES displayed strong correlations with traits relevant to that network such as measured lipids. Penalized regression was also applied to the construction of these network PESs rather than just clumping and thresholding as was undertaken in previous PES studies.[18–20] These penalized regression PESs did explain more variance in most of the networks considering the training set compared to clumping and thresholding, although selecting the appropriate constraint and tuning parameters when many combinations perform similarly is an ongoing challenge for such approaches. For example, the best performing *FES* network PES for BIP was derived through penalized regression, but it included only five variants, which is not representative of the overall network. These issues highlight the need for future study in the construction of PESs, particularly as popular genome-wide approaches for PRSs like LDpred and SBayesR would need to be methodologically adapted for a local gene set or network implemention.[15] The proportion of phenotypic variance explained by PESs derived using these networks could also be boosted in the future by incorporating rare and structural variation, as well as reweighting effect sizes informed by functional annotation. In our previous hypothesis-free screen of pharmagenic enrichment in schizophrenia, we observed networks related to GABAergic and cholinergic signalling.[18] The focused network-based approach used in the current study could be used to capture pharmagenic enrichment scores for existing antipsychotics to enable their application in precision medicine. Future study could explicitly test whether PES profiles in treatment-orientated networks are associated with treatment-response-related variables.

While there is still work needed to confirm that the PES can effectively triage an individual's suitability for drug-repurposing candidates in psychiatry and other disorders, this study represents a key methodological advance as it predicts the desired direction of effect needed to modulate a given target within the context of a PES. Previously, it was not clear whether agonism or antagonism of genes in the set would be clinically useful. Randomized placebo control trials of the PES approach would be a further step to demonstrate its utility and could involve repurposed drugs stratified by the relevant PES or more complex study designs, such as the multi-crossover "*N*-of-one" approach.[69,70] In summary, we present a novel framework to inform precision drug repurposing in psychiatry that could account for individual-level heterogeneity in genetic risk factors, and therefore, improve patient outcomes.

Data and code availability

The analysis code for this study can be found in the following GitHub repository: https://github.com/Williamreay/Directional_anchor_gene_psyciatric_PES. Researchers can access the full UK Biobank data upon approval (https://www.ukbiobank.ac.uk/enable-your-research/apply-for-access). The ASRB cohort is available upon reasonable request and ethics approval (https://www.neura.edu.au/discovery-portal/asrb/). The remaining data are all publicly available, as outlined in the studies cited at relevant positions throughout the main text.

Supplemental information

Supplemental information can be found online at https://doi.org/10.1016/j.ajhg.2022.07.011.

Acknowledgments

This work was supported by a National Health and Medical Research Council (NHMRC) grant (1188493). M.J.C. is supported by an NHMRC Senior Research Fellowship (1121474) (https://www.nhmrc.gov.au/). The funders had no role in study design, data collection and analysis, decision to publish, or preparation of the paper. W.R.R was supported by an Australian Government Research Training Program Stipend. This research was conducted using the UK Biobank resource under the application 58432.

Declaration of interests

W.R.R. and M.J.C. have filed a patent related to the use of the pharmagenic enrichment score framework in complex disorders (WIPO Patent Application WO/2020/237314), an approach used in a section of this study. W.R.R. and M.J.C. also are directors of a company that aims to commercialize the pharmagenic enrichment score platform (PolygenRx Pty Ltd).

Received: May 11, 2022
Accepted: July 13, 2022
Published: September 1, 2022

References

1. Rush, A.J., Trivedi, M.H., Wisniewski, S.R., Nierenberg, A.A., Stewart, J.W., Warden, D., Niederehe, G., Thase, M.E., Lavori, P.W., Lebowitz, B.D., et al. (2006). Acute and longer-term outcomes in depressed outpatients requiring one or several treatment steps: a STAR*D report. Am. J. Psychiatry *163*, 1905–1917.
2. Wimberley, T., Støvring, H., Sørensen, H.J., Horsdal, H.T., MacCabe, J.H., and Gasse, C. (2016). Predictors of treatment resistance in patients with schizophrenia: a population-based cohort study. Lancet Psychiatr. *3*, 358–366.
3. Souery, D., Oswald, P., Massat, I., Bailer, U., Bollen, J., Demyttenaere, K., Kasper, S., Lecrubier, Y., Montgomery, S., Serretti, A., et al. (2007). Clinical factors associated with treatment resistance in major depressive disorder: results from a European multicenter study. J. Clin. Psychiatry *68*, 1062–1070.
4. Hyman, S.E. (2012). Revolution stalled. Sci. Transl. Med. *4*, 155cm11.
5. (2007). Psychiatric drug discovery on the couch. Nat. Rev. Drug Discov. 6, 171.
6. Pushpakom, S., Iorio, F., Eyers, P.A., Escott, K.J., Hopper, S., Wells, A., Doig, A., Guilliams, T., Latimer, J., McNamee, C., et al. (2019). Drug repurposing: progress, challenges and recommendations. Nat. Rev. Drug Discov. *18*, 41–58.
7. Reay, W.R., and Cairns, M.J. (2021). Advancing the use of genome-wide association studies for drug repurposing. Nat. Rev. Genet. *22*, 658–671.
8. Fava, M. (2018). The promise and challenges of drug repurposing in psychiatry. World Psychiatr. *17*, 28–29.
9. Schizophrenia Working Group of the Psychiatric Genomics Consortium, Ripke, S., Walters, J.T., and O'Donovan, M.C. (2020). Mapping genomic loci prioritises genes and implicates synaptic biology in schizophrenia. Preprint at medRxiv. https://doi.org/10.1101/2020.09.12.20192922.
10. Stahl, E.A., Breen, G., Forstner, A.J., McQuillin, A., Ripke, S., Trubetskoy, V., Mattheisen, M., Wang, Y., Coleman, J.R.I., Gaspar, H.A., et al. (2019). Genome-wide association study identifies 30 loci associated with bipolar disorder. Nat. Genet. *51*, 793–803.
11. Watson, H.J., Yilmaz, Z., Thornton, L.M., Hübel, C., Coleman, J.R.I., Gaspar, H.A., Bryois, J., Hinney, A., Leppä, V.M., Mattheisen, M., et al. (2019). Genome-wide association study identifies eight risk loci and implicates metabo-psychiatric origins for anorexia nervosa. Nat. Genet. *51*, 1207–1214.
12. Demontis, D., Walters, R.K., Martin, J., Mattheisen, M., Als, T.D., Agerbo, E., Baldursson, G., Belliveau, R., Bybjerg-Grauholm, J., Bækvad-Hansen, M., et al. (2019). Discovery of the first genome-wide significant risk loci for attention deficit/hyperactivity disorder. Nat. Genet. *51*, 63–75.
13. Wray, N.R., Ripke, S., Mattheisen, M., Trzaskowski, M., Byrne, E.M., Abdellaoui, A., Adams, M.J., Agerbo, E., Air, T.M., Andlauer, T.M.F., et al. (2018). Genome-wide association analyses identify 44 risk variants and refine the genetic architecture of major depression. Nat. Genet. *50*, 668–681.
14. International Schizophrenia Consortium, Purcell, S.M., Wray, N.R., Stone, J.L., Visscher, P.M., O'Donovan, M.C., Sullivan, P.F., and Sklar, P. (2009). Common polygenic variation contributes to risk of schizophrenia and bipolar disorder. Nature *460*, 748–752.
15. Ni, G., Zeng, J., Revez, J.A., Wang, Y., Zheng, Z., Ge, T., Restuadi, R., Kiewa, J., Nyholt, D.R., Coleman, J.R.I., et al. (2021). A Comparison of Ten Polygenic Score Methods for Psychiatric Disorders Applied Across Multiple Cohorts. Biol. Psychiatr. *90*, 611–620. S0006322321012634.
16. Gaspar, H.A., and Breen, G. (2017). Drug enrichment and discovery from schizophrenia genome-wide association results: an analysis and visualisation approach. Sci. Rep. *7*, 12460.
17. Reay, W.R., and Cairns, M.J. (2020). The role of the retinoids in schizophrenia: genomic and clinical perspectives. Mol. Psychiatry *25*, 706–718.
18. Reay, W.R., Atkins, J.R., Carr, V.J., Green, M.J., and Cairns, M.J. (2020). Pharmacological enrichment of polygenic risk for precision medicine in complex disorders. Sci. Rep. *10*, 879.
19. Reay, W.R., El Shair, S.I., Geaghan, M.P., Riveros, C., Holliday, E.G., McEvoy, M.A., Hancock, S., Peel, R., Scott, R.J., Attia, J.R., and Cairns, M.J. (2021). Genetic association and causal inference converge on hyperglycaemia as a modifiable factor to improve lung function. Elife *10*, e63115.
20. Reay, W.R., Geaghan, M.P., 23andMe Research Team, Agee, M., Alipanahi, B., Bell, R.K., Bryc, K., Elson, S.L., Fontanillas, P., Furlotte, N.A., et al. (2022). The genetic architecture of pneumonia susceptibility implicates mucin biology and a relationship with psychiatric illness. Nat. Commun. *13*, 3756.
21. Reay, W.R., Kiltschewskij, D.J., Geaghan, M.P., Atkins, J.R., Carr, V.J., Green, M.J., and Cairns, M.J. (2022). Genetic estimates of correlation and causality between blood-based biomarkers and psychiatric disorders. Sci. Adv. *8*, eabj8969.
22. Gusev, A., Ko, A., Shi, H., Bhatia, G., Chung, W., Penninx, B.W.J.H., Jansen, R., de Geus, E.J.C., Boomsma, D.I., Wright, F.A., et al. (2016). Integrative approaches for large-scale transcriptome-wide association studies. Nat. Genet. *48*, 245–252.
23. Gandal, M.J., Zhang, P., Hadjimichael, E., Walker, R.L., Chen, C., Liu, S., Won, H., van Bakel, H., Varghese, M., Wang, Y., et al. (2018). Transcriptome-wide isoform-level dysregulation in ASD, schizophrenia, and bipolar disorder. Science *362*, eaat8127.
24. Wingo, A.P., Liu, Y., Gerasimov, E.S., Gockley, J., Logsdon, B.A., Duong, D.M., Dammer, E.B., Robins, C., Beach, T.G., Reiman, E.M., et al. (2021). Integrating human brain proteomes with genome-wide association data implicates new proteins in Alzheimer's disease pathogenesis. Nat. Genet. *53*, 143–146.
25. Zhang, J., Dutta, D., Köttgen, A., Tin, A., Schlosser, P., Grams, M.E., Harvey, B., CKDGen Consortium, Yu, B., Boerwinkle, E., et al. (2022). Plasma Proteome analyses in individuals of European and African ancestry identify *cis*-pQTLs and Models for Proteome-wide Association Studies. Nat. Genetics *54*, 593–602.
26. Pain, O., Pocklington, A.J., Holmans, P.A., Bray, N.J., O'Brien, H.E., Hall, L.S., Pardiñas, A.F., O'Donovan, M.C., Owen, M.J., and Anney, R. (2019). Novel Insight Into the Etiology of Autism Spectrum Disorder Gained by Integrating Expression Data With Genome-wide Association Statistics. Biol. Psychiatry *86*, 265–273.
27. Mancuso, N., Freund, M.K., Johnson, R., Shi, H., Kichaev, G., Gusev, A., and Pasaniuc, B. (2019). Probabilistic fine-mapping of transcriptome-wide association studies. Nat. Genet. *51*, 675–682.

28. Giambartolomei, C., Vukcevic, D., Schadt, E.E., Franke, L., Hingorani, A.D., Wallace, C., and Plagnol, V. (2014). Bayesian test for colocalisation between pairs of genetic association studies using summary statistics. PLoS Genet. *10*, e1004383.
29. Zheng, J., Haberland, V., Baird, D., Walker, V., Haycock, P.C., Hurle, M.R., Gutteridge, A., Erola, P., Liu, Y., Luo, S., et al. (2020). Phenome-wide Mendelian randomization mapping the influence of the plasma proteome on complex diseases. Nat. Genet. *52*, 1122–1131.
30. de Klein, N., Tsai, E.A., Vochteloo, M., Baird, D., Huang, Y., Chen, C.-Y., van Dam, S., Deelen, P., Bakker, O.B., Garwany, O.E., et al. (2021). Brain expression quantitative trait locus and network analysis reveals downstream effects and putative drivers for brain-related diseases. Preprint at bioRxiv. https://doi.org/10.1101/2021.03.01.433439.
31. Robins, C., Liu, Y., Fan, W., Duong, D.M., Meigs, J., Harerimana, N.V., Gerasimov, E.S., Dammer, E.B., Cutler, D.J., Beach, T.G., et al. (2021). Genetic control of the human brain proteome. Am. J. Hum. Genet. *108*, 400–410.
32. Võsa, U., Claringbould, A., Westra, H.-J., Bonder, M.J., Deelen, P., Zeng, B., Kirsten, H., Saha, A., Kreuzhuber, R., Kasela, S., et al. (2018). Large-scale cis- and trans-eQTL analyses identify thousands of genetic loci and polygenic scores that regulate blood gene expression. Nat. Genetics *53*, 1300–1310.
33. Hemani, G., Zheng, J., Elsworth, B., Wade, K.H., Haberland, V., Baird, D., Laurin, C., Burgess, S., Bowden, J., Langdon, R., et al. (2018). The MR-Base platform supports systematic causal inference across the human phenome. Elife *7*, e34408.
34. Freshour, S., Kiwala, S., Cotto, K.C., Coffman, A.C., McMichael, J.F., Song, J., Griffith, M., Griffith, O.L., and Wagner, A.H. (2020). Integration of the Drug-Gene Interaction Database (DGIdb) with open crowdsource efforts. Bioinformatics *49*, D1144–D1151.
35. Szklarczyk, D., Gable, A.L., Lyon, D., Junge, A., Wyder, S., Huerta-Cepas, J., Simonovic, M., Doncheva, N.T., Morris, J.H., Bork, P., et al. (2019). STRING v11: protein-protein association networks with increased coverage, supporting functional discovery in genome-wide experimental datasets. Nucleic Acids Res. *47*, D607–D613.
36. Raudvere, U., Kolberg, L., Kuzmin, I., Arak, T., Adler, P., Peterson, H., and Vilo, J. (2019). g:Profiler: a web server for functional enrichment analysis and conversions of gene lists (2019 update). Nucleic Acids Res. *47*, W191–W198.
37. de Leeuw, C.A., Mooij, J.M., Heskes, T., and Posthuma, D. (2015). MAGMA: generalized gene-set analysis of GWAS data. PLoS Comput. Biol. *11*, e1004219.
38. Reay, W.R., Geaghan, M.P., 23 and Me Research Team, and Cairns, M.J. (2021). Genome-wide meta-analysis of pneumonia suggests a role for mucin biology and provides novel drug repurposing opportunities.. Preprint at medRxiv. https://doi.org/10.1101/2021.01.24.21250424.
39. Choi, S.W., and O'Reilly, P.F. (2019). PRSice-2: Polygenic Risk Score software for biobank-scale data. GigaScience *8*, giz082.
40. Mak, T.S.H., Porsch, R.M., Choi, S.W., Zhou, X., and Sham, P.C. (2017). Polygenic scores via penalized regression on summary statistics. Genet. Epidemiol. *41*, 469–480.
41. Loughland, C., Draganic, D., McCabe, K., Richards, J., Nasir, A., Allen, J., Catts, S., Jablensky, A., Henskens, F., Michie, P., et al. (2010). Australian Schizophrenia Research Bank: a database of comprehensive clinical, endophenotypic and genetic data for aetiological studies of schizophrenia. Aust. N. Z. J. Psychiatry *44*, 1029–1035.
42. Lee, S.H., Goddard, M.E., Wray, N.R., and Visscher, P.M. (2012). A better coefficient of determination for genetic profile analysis. Genet. Epidemiol. *36*, 214–224.
43. Aschard, H., Vilhjálmsson, B.J., Joshi, A.D., Price, A.L., and Kraft, P. (2015). Adjusting for heritable covariates can bias effect estimates in genome-wide association studies. Am. J. Hum. Genet. *96*, 329–339.
44. Martin, J., Khramtsova, E.A., Goleva, S.B., Blokland, G.A.M., Traglia, M., Walters, R.K., Hübel, C., Coleman, J.R.I., Breen, G., Børglum, A.D., et al. (2021). Examining Sex-Differentiated Genetic Effects Across Neuropsychiatric and Behavioral Traits. Biol. Psychiatry *89*, 1127–1137.
45. Wainberg, M., Sinnott-Armstrong, N., Mancuso, N., Barbeira, A.N., Knowles, D.A., Golan, D., Ermel, R., Ruusalepp, A., Quertermous, T., Hao, K., et al. (2019). Opportunities and challenges for transcriptome-wide association studies. Nat. Genet. *51*, 592–599.
46. Lipton, S.A., Choi, Y.-B., Takahashi, H., Zhang, D., Li, W., Godzik, A., and Bankston, L.A. (2002). Cysteine regulation of protein function–as exemplified by NMDA-receptor modulation. Trends Neurosci. *25*, 474–480.
47. Yawalkar, R., Changotra, H., and Gupta, G.L. (2018). Protective influences of N-acetylcysteine against alcohol abstinence-induced depression by regulating biochemical and GRIN2A, GRIN2B gene expression of NMDA receptor signaling pathway in rats. Neurochem. Int. *118*, 73–81.
48. Neumeyer, S., Hemani, G., and Zeggini, E. (2020). Strengthening Causal Inference for Complex Disease Using Molecular Quantitative Trait Loci. Trends Mol. Med. *26*, 232–241.
49. Meng, F., Xi, Y., Huang, J., and Ayers, P.W. (2021). A curated diverse molecular database of blood-brain barrier permeability with chemical descriptors. Sci. Data *8*, 289.
50. Sunitha, K., Hemshekhar, M., Thushara, R.M., Santhosh, M.S., Yariswamy, M., Kemparaju, K., and Girish, K.S. (2013). N-Acetylcysteine amide: a derivative to fulfill the promises of N-Acetylcysteine. Free Radic. Res. *47*, 357–367.
51. Bauer, T.M., Shaw, A.T., Johnson, M.L., Navarro, A., Gainor, J.F., Thurm, H., Pithavala, Y.K., Abbattista, A., Peltz, G., and Felip, E. (2020). Brain Penetration of Lorlatinib: Cumulative Incidences of CNS and Non-CNS Progression with Lorlatinib in Patients with Previously Treated ALK-Positive Non-Small-Cell Lung Cancer. Target. Oncol. *15*, 55–65.
52. Avellini, L., Terracina, L., and Gaiti, A. (1994). Linoleic acid passage through the blood-brain barrier and a possible effect of age. Neurochem. Res. *19*, 129–133.
53. Jiang, Y.Y., Shui, J.C., Zhang, B.X., Chin, J.W., and Yue, R.S. (2020). The Potential Roles of Artemisinin and Its Derivatives in the Treatment of Type 2 Diabetes Mellitus. Front. Pharmacol. *11*, 585487.
54. Kuhn, M., Letunic, I., Jensen, L.J., and Bork, P. (2016). The SIDER database of drugs and side effects. Nucleic Acids Res. *44*, D1075–D1079.
55. Wishart, D.S., Feunang, Y.D., Guo, A.C., Lo, E.J., Marcu, A., Grant, J.R., Sajed, T., Johnson, D., Li, C., Sayeeda, Z., et al. (2018). DrugBank 5.0: a major update to the DrugBank database for 2018. Nucleic Acids Res. *46*, D1074–D1082.
56. Nelson, S.D., and Munger, M.A. (2013). Icosapent Ethyl for Treatment of Elevated Triglyceride Levels. Ann. Pharmacother. *47*, 1517–1523.

57. Nery, F.G., Li, W., DelBello, M.P., and Welge, J.A. (2021). N-acetylcysteine as an adjunctive treatment for bipolar depression: A systematic review and meta-analysis of randomized controlled trials. Bipolar Disord. *23*, 707–714.
58. Lerner, V., Miodownik, C., Gibel, A., Sirota, P., Bush, I., Elliot, H., Benatov, R., and Ritsner, M.S. (2013). The retinoid X receptor agonist bexarotene relieves positive symptoms of schizophrenia: a 6-week, randomized, double-blind, placebo-controlled multicenter trial. J. Clin. Psychiatry *74*, 1224–1232.
59. Stanicka, J., Rieger, L., O'Shea, S., Cox, O., Coleman, M., O'Flanagan, C., Addario, B., McCabe, N., Kennedy, R., and O'Connor, R. (2018). FES-related tyrosine kinase activates the insulin-like growth factor-1 receptor at sites of cell adhesion. Oncogene *37*, 3131–3150.
60. Javitt, D.C., Silipo, G., Cienfuegos, A., Shelley, A.M., Bark, N., Park, M., Lindenmayer, J.P., Suckow, R., and Zukin, S.R. (2001). Adjunctive high-dose glycine in the treatment of schizophrenia. Int. J. Neuropsychopharmacol. *4*, 385–391.
61. Yolland, C.O., Hanratty, D., Neill, E., Rossell, S.L., Berk, M., Dean, O.M., Castle, D.J., Tan, E.J., Phillipou, A., Harris, A.W., et al. (2020). Meta-analysis of randomised controlled trials with *N* -acetylcysteine in the treatment of schizophrenia. Aust. N. Z. J. Psychiatry *54*, 453–466.
62. Nasir, M., and Bloch, M.H. (2019). Trim the fat: the role of omega-3 fatty acids in psychopharmacology. Ther. Adv. Psychopharmacol. *9*. 2045125319869791.
63. Moon, A.L., Haan, N., Wilkinson, L.S., Thomas, K.L., and Hall, J. (2018). CACNA1C: Association With Psychiatric Disorders, Behavior, and Neurogenesis. Schizophr. Bull. *44*, 958–965.
64. Okorji, U.P., Velagapudi, R., El-Bakoush, A., Fiebich, B.L., and Olajide, O.A. (2016). Antimalarial Drug Artemether Inhibits Neuroinflammation in BV2 Microglia Through Nrf2-Dependent Mechanisms. Mol. Neurobiol. *53*, 6426–6443.
65. Chen, W., Jin, D., Shi, Y., Zhang, Y., Zhou, H., and Li, G. (2020). The underlying mechanisms of lorlatinib penetration across the blood-brain barrier and the distribution characteristics of lorlatinib in the brain. Cancer Med. *9*, 4350–4359.
66. Hjelm, B.E., Rollins, B., Mamdani, F., Lauterborn, J.C., Kirov, G., Lynch, G., Gall, C.M., Sequeira, A., and Vawter, M.P. (2015). Evidence of Mitochondrial Dysfunction within the Complex Genetic Etiology of Schizophrenia. Mol. Neuropsychiatry *1*, 201–219.
67. Gold, L., Ayers, D., Bertino, J., Bock, C., Bock, A., Brody, E.N., Carter, J., Dalby, A.B., Eaton, B.E., Fitzwater, T., et al. (2010). Aptamer-based multiplexed proteomic technology for biomarker discovery. PLoS One *5*, e15004.
68. Rohloff, J.C., Gelinas, A.D., Jarvis, T.C., Ochsner, U.A., Schneider, D.J., Gold, L., and Janjic, N. (2014). Nucleic Acid Ligands With Protein-like Side Chains: Modified Aptamers and Their Use as Diagnostic and Therapeutic Agents. Mol. Ther. Nucleic Acids *3*, e201.
69. Schork, N.J. (2015). Personalized medicine: Time for one-person trials. Nature *520*, 609–611.
70. Percha, B., Baskerville, E.B., Johnson, M., Dudley, J.T., and Zimmerman, N. (2019). Designing Robust N-of-1 Studies for Precision Medicine: Simulation Study and Design Recommendations. J. Med. Internet Res. *21*, e12641.

The American Journal of Human Genetics, Volume *109*

Supplemental information

Genetics-informed precision treatment formulation in schizophrenia and bipolar disorder

William R. Reay, Michael P. Geaghan, Joshua R. Atkins, Vaughan J. Carr, Melissa J. Green, and Murray J. Cairns

SUPPLEMENTARY FIGURES

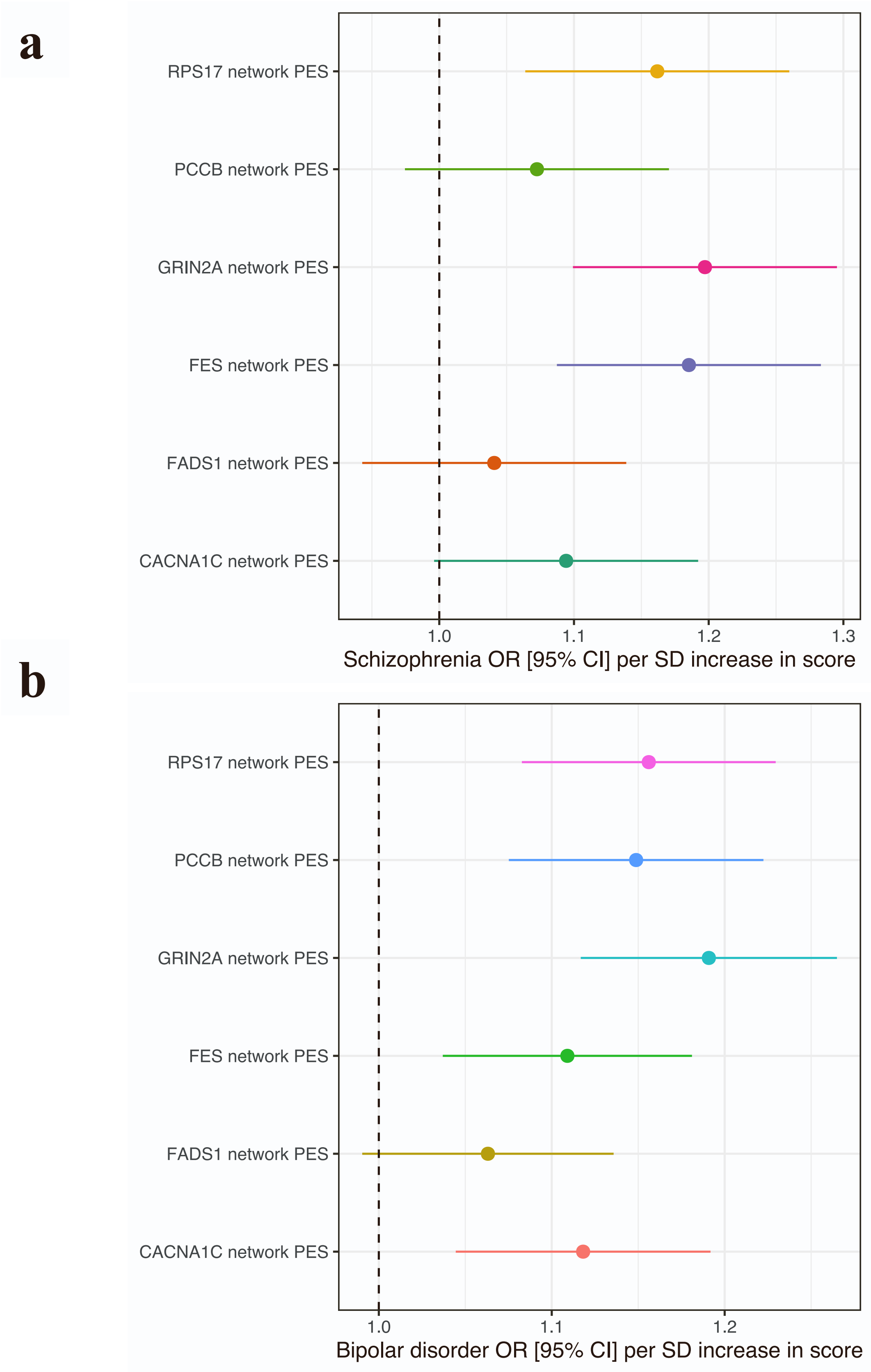

Figure S1. Network PES effect sizes in the training set for schizophrenia and bipolar disorder. Forest plot of odds ratio for schizophrenia (**a**) and bipolar disorder (**b**) per standard deviation increase in each score is presented. Error bars denote 95% confidence intervals.

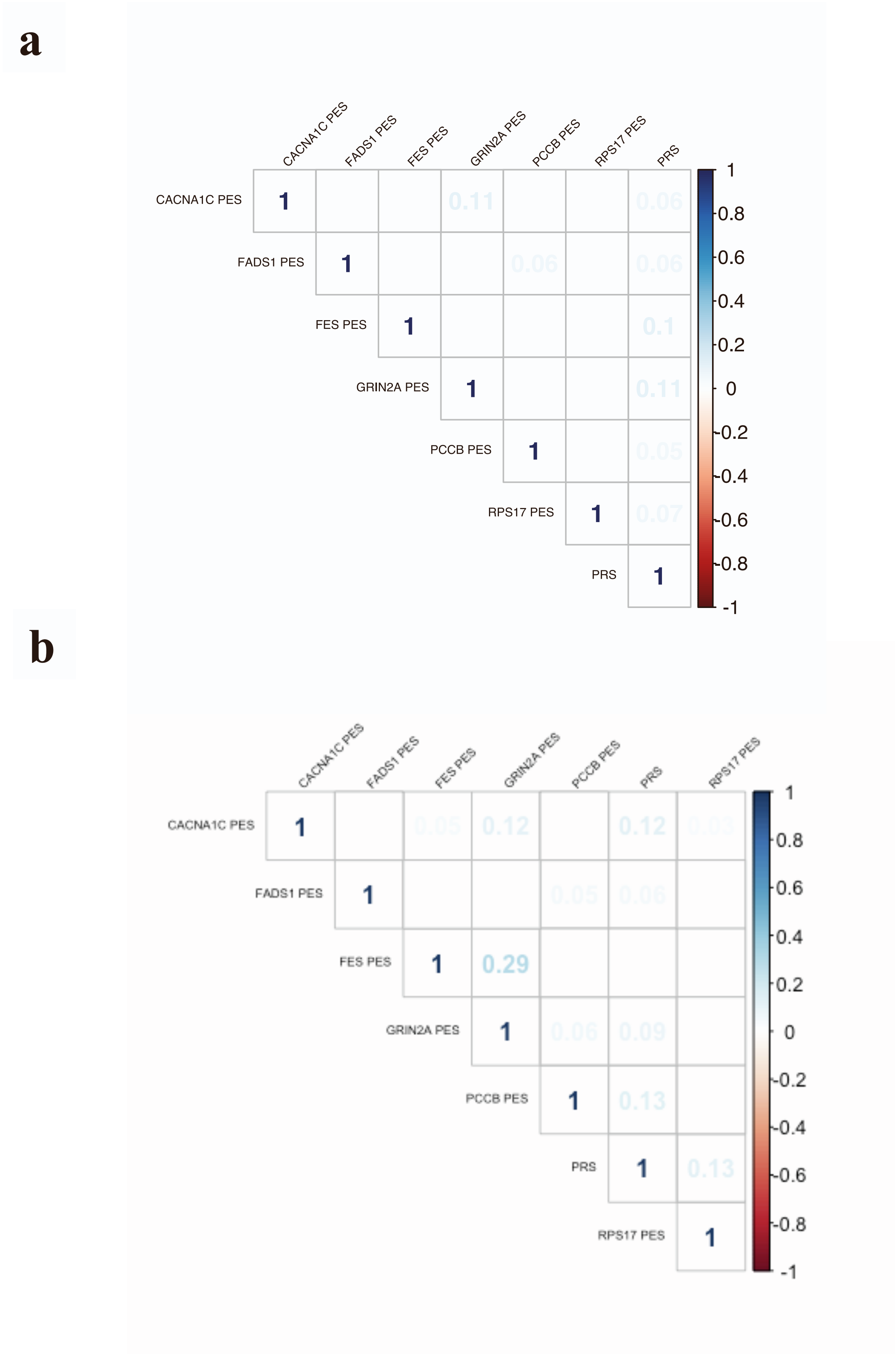

Figure S2. Correlation plot (Pearson's) amongst network PES and genome wide PRS. Nominally significant correlations ($P < 0.05$) have their correlation coefficients displayed. The top panel (**a**) is for schizophrenia scores and the bottom panel (**b**) is for BIP scores.

SUPPLEMENTARY METHODS

FUSION implementation of transcriptome and proteome wide association studies (TWAS/PWAS)

TWAS

Brain related SNP weights (multivariate GReX) TWAS were derived from GTEx v7 and PsychENCODE, whilst whole blood weights were also obtained from GTEx v7 [1, 2]. The GTEx v7 SNP weights comprise data from 11 different brain regions – with the sample sizes of the cohorts utilised for GReX estimation as follows: amygdala (N=88), anterior cingulate cortex (N=109), caudate (N=144), cerebellar hemisphere (N=125), cerebellum (N=154), cortex (N=136), frontal cortex (N=136), hippocampus (N=111), hypothalamus (N=108), nucleus accumbens (N=130), and putamen (N=80). We implemented FUSION using default parameters for these 11 tissues using the HapMap3 SNPs from the 1000 genomes phase 3 European reference panel as a linkage disequilibrium (LD) estimate, to correspond with how the weights were calculated with those same HapMap3 SNPs. The TWAS using whole blood weights utilised the same LD reference strategy, with the number of GTEx v7 participants in these models 369. Frontal or cerebral cortex tissue from the larger PsychENCODE cohort was also utilised in terms of SNP weights, with a sample size of 1695. As described in the original PsychENCODE publication, gene-wise GReX were estimated using all imputed SNPs, not just the HapMap3 panel, and thus, we utilised the full suite of the phase 3 1000 genomes European subset as the LD reference. Summary statistics were also munged using the FOCUS wrapper for the 'munge_sumstats.py', whereby SNPs were retained with an imputation INFO > 0.9, as well as removing indels, strand ambiguous SNPs and SNPs with MAF < 0.01. In total, considering all GReX models that survived the FUSION pipeline's internal QC before calculating the TWAS *Z*, there were 55,834 and 54,986 models tested for SZ and BIP, respectively. This equated to approximately 18,000 unique genes tested for both disorders. However, we conservatively adjusted for all of the models tested in terms of multiple-testing correction to only priortise the most statistically significant signals for further consideration.

PWAS

There were two tissues for which were obtained SNP weights related to protein expression – the dorsolateral prefrontal cortex (DLPFC, N = 376) and plasma (N = 7213) [3, 4]. It should be noted that the plasma weights were derived from the European subset of the study cohort,

and the authors only used the elastic net method to derive GReX. Analogous to the difference between the GTEx v7 and PsychENCODE studies above, the DLPFC SNP weights were estimated using the HapMap3 panel, and thus, we only used those SNPs as an LD reference. The plasma PWAS utilised the full reference panel. The number of protein models available in each tissue for the two GWAS was as follows: SZ – plasma = 484, DLPFC = 1443. BIP – plasma = 350, DLPFC = 1400.

Probabilistic finemapping of TWAS signals which represent a plausible repurposing opportunity (FOCUS)

We sought to finemap the region of genes for which their genetically regulated expression was associated with SZ or BIP after Bonferroni correction in a fashion that could be counteracted by an approved drug [5]. There were two reference panels utilised for finemapping – for genes uncovered from a GTEx v7 tissue, we utilised the default combined FOCUS SNP weight set which collated GTEx v7 tissues, DLPFC (CommonMind), blood (YFS, NTR), and adipose (METSIM) SNP weight sets (https://www.dropbox.com/s/ep3dzlqnp7p8e5j/focus.db?dl=0), with genes discovered using the PsychENCODE weights finemapped specifically using that panel given the different LD parameters and its more complete set of genes with cis-heritable models in that one tissue. The default FOCUS panel was tested by prioritising brain related GReX, followed by predictive accuracy in the absence of a brain tissue SNP-weight set. In SZ, there were twelve such candidates with four genes displaying a high posterior inclusion probability (*PIP*) of over 80% of being in the 90% credible set (*GRIN2A*, *FES, PCCB,* and *CACNA1D*), whilst two additional genes surpassed the more relaxed criterion of *PIP* > 0.4 (*CACNA1C* and *RPS17*). The remaining genes all had very low *PIP*, which suggested they were not a likely causal gene at that locus based on TWAS data (*NEK4*, *MAPK3*, *TLR9*, *CHRNA2, PSMB8, SERPINC1*, and *CTSS*). There were two candidate genes for BIP (*NEK4* and *FADS1*), with only *FADS1* surpassing the relaxed *PIP* threshold of 40% using the PsychENCODE SNP weights.

In the case of candidate gene achieving PIP > 0.8, we also considered the use of a more conservative Bernoulli prior for the models of each causal indicator [$c \sim \text{Bern}(p)$] of $p = 1 \times 10^{-5}$, rather than the default $p = 1 \times 10^{-3}$, with the implemention of these priors outlined more extensively in Mancuso *et al.* [5]. We found this had no large effect on the *PIP* for three of the genes, with the updated *PIP* as follows: *FES* – *PIP* = 1, *GRIN2A*– *PIP* = 0.926, and *PCCB* – *PIP* = 1. The *CACNA1D PIP* was ablated upon using this more conservative prior (*PIP* =

0.478), with more evidence in this construct for *GNL3* as a candidate causal gene for this region (*PIP* = 0.88). As described in the following section, we believe that the complexity of the region in which *CACNA1D* is located and the weakness of its GReX model necessitates removing this gene as a candidate DA-gene.

CACNA1D region

Despite *CACNA1D* exhibiting a high *PIP* using the default prior, we believe that this association is not confident enough to carry forward with as a candidate directional anchor gene. Firstly, whilst this gene displayed a strong association in the PsychENCODE cortical TWAS ($Z = 6.38$), its GReX models only explained a very small amount of phenotypic variance that was only nominally statistically significant relative to the other GReX models considered as candidate DA-genes (cross-validated $R^2 = 0.003$, $P = 0.024$). This GReX model, which performed best using a Bayesian sparse linear mixed model in the PsychENCODE study, was not found in any of the smaller sample size GTEx tissues – suggesting that the effect of *cis*-acting SNPs on expression was very weak for this gene and could only be detected at larger sample sizes. For comparison, the mean cross-validated variance explained in mRNA expression by GReX models of non-MHC genes from the PsychENCODE weight set was approximately 8.64%. We subjected the SNP weights in the *CACNA1D* GReX model and the same signal in the SZ GWAS to colocalisation analyses and found no strong evidence that *CACNA1D* expression was associated with both traits, either driven by a different or the same underlying causal variant ($PP_{H3} = 0.164$, $PP_{H4} = 0.228$), which is likely driven by the fact that the GReX model is only weakly statistically significant in terms of its correlation with expression. Finally, the region in which *CACNA1D* is located on chromosome 3 is a gene-dense region with multiple different biologically plausible causal genes such as *NEK4*, and *GNL3*, which becomes the most likely causal candidate using the more conservative prior, as outlined above.

Selection of eQTL and pQTL as instrumental variables

The selection of QTLs as instrumental variables (IV) can be challenging, particularly due to the onerous assumptions of MR for a valid IV. In most cases, only a single or a handful of IVs can be specified for mRNA or protein expression of any given gene, and, as a result, the suite of sensitivity analyses applied to more polygenic MR cannot be applied such as median, modal, and Egger regression based estimators [6, 7]. This reinforces that only the strongest QTL related signals should be chosen, as well as those associated with further genes such that there

is less possibility for horizontal pleiotropy through the action of another transcript or protein. MR aims to infer a causal relationship and a corresponding causal estimate, which justifies conservatism in its approach. In the following section, we detail how IVs were chosen from the eQTL and pQTL studies we selected for analyses.

Expression quantitative trait loci (eQTL)

An eQTL study from blood (eQTLgen) and brain (MetaBrain) were selected as the source of eQTLs [8, 9]. These studies had larger sample sizes than the cohorts utilised to derive GReX models for TWAS, however, TWAS weights were unavailable in both instances. The eQTLgen study was a meta-analysis of blood-based eQTL cohorts, with a sample size up to 31,684, whilst MetaBrain was also a meta-analysis of brain eQTL cohorts across different regions. We selected three MetaBrain tissues which had a European ancestry sample size > 200, cortex (N = 2970), cerebellum (N= 492), and basal ganglia (N = 208). In both tissue types, blood, and brain, we sequentially applied the following quality control steps

i) Retain only variants associated with mRNA expression using a genome-wide significance threshold ($P < 5 \times 10^{-8}$). This threshold would be over-conservative for eQTL discovery, however, given IVs must be strongly associated with the exposure, we concluded that using only genome-wide significant signals would assist to define the strongest signals.

ii) Variants associated with three or fewer genes at genome-wide significance were retained to attempt to minimise the inclusion of highly pleiotropic signals or LD dense regions where many genes can be implicated.

iii) Identify the most significant independent signals (LD $r^2 < 0.001$) from each 10000 kilobase clump (one megabase), with LD inferred using the 1000 genomes phase 3 reference panel. This is also conservative and reduces the number of genes with suitable IVs available – however, this assists to further refine the strongest signals in terms of statistical significance. It should be noted that statistical significance does not necessarily define the most likely causal variant, with future work required to refine likely causal candidates in eQTL studies with greater precision. As outlined in the main text, our TWAS was discovery orientated, meaning that these MR analyses could afford their conservatism.

The above quality control steps left the following numbers of genes to be tested in each tissue with a suitable IV – blood (N_{Genes} = 2880), cortex (N_{Genes} = 1833), cerebellum (N_{Genes} = 1162), and basal ganglia (N_{Genes} = 375).

Protein quantitative trait loci (pQTL)

As in the eQTL analyses we used both blood and brain pQTLs [3, 7]. The blood pQTL were assembled from the "tier 1" pQTLs from Zheng *et al.*, as outlined fully in that study. Briefly, pQTL results from five studies were collated and designated a level of confidence using a tiered system – whereby tier 1, the highest level of confidence, consisted of genome-wide pQTLs that were associated with less than five genes as well as exhibiting consistent effects (based on heterogeneity and colocalisation) across contributing studies they were available in. We further filtered these variants by retaining only those associated with three or fewer genes at genome-wide significance, as well as gene-wise filtering of any dependent SNPs (LD $r^2 > 0.001$). The LD selection step for the pQTLs was slightly less stringent than what was applied to the eQTLs as it was performed per gene rather than per one megabase clump. This was because there were significantly fewer pQTL to begin with, and many genes did not have their protein levels assayed in the contributing studies. There were 891 genes remaining after the above analysis with a valid pQTL. A small number of *trans* pQTL were included in the tier 1 category, which we retained for discovery purposes but did not include any of these genes as final candidate directional anchor genes. The ROSMAP DLPFC cohort contributed the brain pQTL (N = 376), which was the same cohort utilised for estimating the PWAS weights. We also retained only genome-wide significant pQTLs that were also at least nominally significant ($P < 0.05$) in a smaller sample size replication cohort from the Banner Sun Health Research group (N = 189). Thereafter, SNPs were retained if they were associated with three or fewer genes, with the same gene-wise clumping applied as was the case in the blood pQTL studies. In total, there were 479 DLPFC genes with a valid pQTL.

Sensitivity analyses for candidate target genes from Mendelian randomisation

There were three candidate directional anchor genes from the MR analyses that could represent repurposing opportunities for schizophrenia (*PCCB*, *NEK1*, and *PTK2B*), whilst there were two for bipolar disorder (*FADS1* and *MAP2K2*). All of these genes were found using eQTLs as IVs, except for *MAP2K2*. Given that the *MAP2K2* IV was a *trans*-pQTL, we decided not to include this gene as a candidate directional anchor gene for a PES network, however, future study to refine the mechanism by which the IV SNP acts in *trans* to regulate protein expression

could render this gene as a suitable repurposing opportunity. For the remaining genes we considered the following three factors as sensitivity analyses:

i) The genomic location of the IV SNP relative to the gene of interest. IVs located within or proximal to the gene of interest could be viewed as stronger evidence, although genic proximity does not guarantee causality.

ii) Colocalisation between expression of the gene and schizophrenia or bipolar disorder

iii) Phenome-wide MR (MR-pheWAS) using SNP effects from the IEUGWAS db. Specifically, we harmonised the IV effect allele to that of the other GWAS in the database and calculated a Wald ratio, along with its corresponding standard error and *P* value, to define other phenotypes for which expression of that gene could be causally implicated. These data were also helpful to refine other genes which could be associated with the IV.

It should be noted that all of the candidate genes only had a single IV, and, as a result, conventional polygenic MR sensitivity analyses like models with other IV assumptions (majority valid, plurality valid, and InSIDE) could not be implemented. We outline the results for the sensitivity analyses and MR-pheWAS for each gene below.

PCCB

The IV SNP for *PCCB* was an eQTL from the MetaBrain cortex meta-analysis (rs480330), and is intronic within the *PCCB* gene itself, supporting its relevance for that gene. Colocalisation analyses using default priors implicated that *PCCB* mRNA expression and schizophrenia were both associated, however, there was evidence of a different underlying causal variant (PP_{H3} = 0.95. Interestingly, if the prior probability for a shared causal variant is increased from the default of 1 x 10^{-5} to 1 x 10^{-5}, there is a corresponding rise in the posterior probability of a shared underlying causal variant which approaches 40%. The MR-pheWAS revealed several interesting potential associations which support that increased cortical expression *PCCB* could be protective for schizophrenia, for instance, an association with several other psychiatric phenotypes as outlined in the main text. Expression of *PCCB* was linked to some circulating biochemical factors which could plausibly mediate some of its relationship with schizophrenia, such as sex-hormone binding globulin, testosterone, and bilirubin. Full results of these analyses are presented in supplementary table 11. Importantly, the strongest association was with *PCCB* expression, rather than other genes, with the *PCCB* eQTL data in the IEUGWAS database from blood.

NEK1

There was moderate evidence of a different underlying causal variant (PP_{H3} = 0.76, PP_{H4} = 0.24), although the evidence for H_4 (shared causal variant) does trend towards 0.8 if you have a larger prior probability for that hypothesis > 1 x 10^{-5}. The IV for this gene was intergenic and associated with three other genes at phenome-wide significance, including more significantly with the close-by gene *SH3FR1*, therefore, this association should be treated cautiously. Moreover, this gene did not survive correction in the TWAS but trended towards it $Z = 3.43$, $P = 6.14 \times 10^{-4}$. We decided that this signal could not be fully resolved given that the IV was associated with the expression of another proximal gene with more significance than *NEK1*.

PTK2B

There was strong evidence of a different underlying causal variant (PP_{H3} = 1), remaining consistent with a larger prior probability for H_4. The IV SNP for this gene is intronic within the nearby *TRIM35* and the association of this gene with SZ via MR is larger, therefore, this is a lower confidence association with *PTK2B*.

FADS1

There was strong evidence of a different underlying causal variant between *FADS1* expression and bipolar, although both were associated (PP_{H3} = 1). The IV SNP is a non-coding transcript exon variant for the *FADS1* proximal *MYRF* gene, however, the MR-pheWAS supports a strong effect on lipid biology consistent with this gene and nearby *FADS2* (Supplementary Table 12), as does the TWAS finemapping relative to *MYRF*. Given this gene also had support from TWAS, and a more biologically consistent MR-pheWAS result, we selected this as a candidate directional anchor gene.

UK Biobank cohort sample specification and phenotype definition

The UK Biobank is a large prospective cohort of approximately 500,000 participants for which extensive clinical, phenotypic, genetic, and molecular data are available [10]. Our group has previously processed the SNP array data through a series of quality control steps to retain a final cohort with 336,896 unrelated participants with white British ancestry for which 13,568,914 high quality (INFO > 0.8 or physically genotyped, missingness < 2%, no strong

Hardy-Weinberg deviations) variants were available with a minimum minor allele frequency of 0.001%. Full details of the quality control applied are outlined in Reay *et al.* [11].

We utilised an integrative strategy for defining cases of schizophrenia and bipolar disorder. Given the diagnostic uncertainty and heterogeneity associated with these two disorders, we decided to implement a relatively broad phenotyping approach, however, given the discovery orientated nature of these analyses we feel that this is warranted. Specifically, we utilised three lines of evidence to define a schizophrenia or bipolar disorder diagnosis – self-reported doctor diagnosis at the assessment interview, linked primary or secondary ICD-10 inpatient records, and responses to the mental health questionnaire (MHQ) question regarding what mental illness a person had ever been diagnosed with by a psychiatrist. We selected individuals who satisfied any of these criteria and detail the specific phenotype codes below, note that individuals will satisfy multiple criteria in many instances, so the total is not a sum of each category, rather anyone who appears in at least one of the categories.

Schizophrenia:

- Self-reported at baseline (field 20002, code = 1289) – N = 334
- ICD-10 primary of secondary (field 41202 and 41205, codes = F20.1-9) – N = 453
- Mental health questionnaire (of 110,278 individuals in our genotype cohort who completed) – N = 90
- Total number of cases = 631

Bipolar disorder:

- Self-reported at baseline (field 20002, code = 1291) – N = 921
- ICD-10 primary of secondary (field 41202 and 41205, codes = F31.1-9) – N = 915
- Mental health questionnaire (of 110,278 individuals in our genotype cohort who completed) – N = 546
- N = 485 individuals self-reported at baseline and were hospitalized with a relevant ICD-10 code.
- Total number of cases = 1657
-

Controls were defined using the mental health questionnaire data also. These participants were 75,201 European, unrelated individuals with genotype data available which completed the MHQ and did not self-report any mental health conditions and did not meet any of the baseline

SZ or BIP conditions. Controls were randomly selected from this cohort of 75,201 participants such that there were double the number to that of cases in the schizophrenia, bipolar disorder training, and bipolar disorder test set. The controls used in each of these three cohorts were independent of one another. In the case of the penalised regression (lassosum) models we used the controls from the opposite disorder as the LD reference, that is, for the schizophrenia cohort, the independent bipolar training cohort controls were used to account for LD in the regularization process. In the schizophrenia training cohort (N_{Cases} = 631, $N_{Controls}$ = 1262) the schizophrenia cases were 63.2% male with a mean age of 55.4 (SD = 8.14), whilst the controls were majority female (51.8%) and older with a mean age of 56.6 (SD = 7.58). The BIP training set, which was 70% of the UKBB cases and double the number of randomly selected controls (N_{Cases} = 1161, $N_{Controls}$ = 2322), had cases (56.8%) and controls (52%) which were majority female, a younger mean age of cases (55.6 years old vs 56.3 years old). The composition of the BIP training set (N_{Cases} = 496, $N_{Controls}$ = 992) was similar: cases – 56.3% female, mean age = 55.6 years old (SD = 8.11); controls – 51.6% female, mean age = 56.7 years (SD = 7.63). In terms of genotyping batch, no more than 2% of any of the cohorts were from any one batch, however, batch was still included as a downstream covariate for stringency.

Australian Schizophrenia Research Bank (ASRB) cohort

The ASRB cohort in terms of genotyping and quality control has been described extensively previously [12]. Briefly, the ASRB was deeply phenotype cohort of recruited cases and non-neuropsychiatric controls as outlined by Loughland *et al.* [13]. The ASRB was used at the validation cohort for schizophrenia due to the comparatively small number of cases in the UKBB, however, given the ASRB was part of the schizophrenia GWAS, we retrained the models for replication in the UKBB training cohort with summary statistics that had the ASRB removed. After quality control, there were 425 cases (66.6% male, mean age = 39.88 years old (SD = 10.92) and 251 controls (56.2% female, mean age = 39.50 years old (SD = 13.4). There were 7,154,884 variants in this cohort available for analysis after post-imputation filtering.

SUPPLEMENTARY REFERENCES

1. Gandal MJ, Zhang P, Hadjimichael E, Walker RL, Chen C, Liu S, et al. Transcriptome-wide isoform-level dysregulation in ASD, schizophrenia, and bipolar disorder. Science. 2018;362.
2. Gusev A, Ko A, Shi H, Bhatia G, Chung W, Penninx BWJH, et al. Integrative approaches for large-scale transcriptome-wide association studies. Nat Genet. 2016;48:245–252.
3. Wingo AP, Liu Y, Gerasimov ES, Gockley J, Logsdon BA, Duong DM, et al. Integrating human brain proteomes with genome-wide association data implicates new proteins in Alzheimer's disease pathogenesis. Nat Genet. 2021;53:143–146.
4. Zhang J, Dutta D, Köttgen A, Tin A, Schlosser P, Grams ME, et al. Large Bi-Ethnic Study of Plasma Proteome Leads to Comprehensive Mapping of *cis* -pQTL and Models for Proteome-wide Association Studies. Genetics; 2021.
5. Mancuso N, Freund MK, Johnson R, Shi H, Kichaev G, Gusev A, et al. Probabilistic fine-mapping of transcriptome-wide association studies. Nat Genet. 2019;51:675–682.
6. Reay WR, Cairns MJ. Advancing the use of genome-wide association studies for drug repurposing. Nat Rev Genet. 2021. 23 July 2021. https://doi.org/10.1038/s41576-021-00387-z.
7. Zheng J, Haberland V, Baird D, Walker V, Haycock PC, Hurle MR, et al. Phenome-wide Mendelian randomization mapping the influence of the plasma proteome on complex diseases. Nat Genet. 2020. 7 September 2020. https://doi.org/10.1038/s41588-020-0682-6.
8. Võsa U, Claringbould A, Westra H-J, Bonder MJ, Deelen P, Zeng B, et al. Unraveling the polygenic architecture of complex traits using blood eQTL metaanalysis. Genomics; 2018.
9. de Klein N, Tsai EA, Vochteloo M, Baird D, Huang Y, Chen C-Y, et al. Brain expression quantitative trait locus and network analysis reveals downstream effects and putative drivers for brain-related diseases. Genomics; 2021.
10. Bycroft C, Freeman C, Petkova D, Band G, Elliott LT, Sharp K, et al. The UK Biobank resource with deep phenotyping and genomic data. Nature. 2018;562:203–209.
11. Reay WR, Geaghan MP, 23andMe Research Team, Cairns MJ. Genome-wide meta-analysis of pneumonia suggests a role for mucin biology and provides novel drug repurposing opportunities. Respiratory Medicine; 2021.
12. Reay WR, Atkins JR, Carr VJ, Green MJ, Cairns MJ. Pharmacological enrichment of polygenic risk for precision medicine in complex disorders. Sci Rep. 2020;10:879.
13. Loughland C, Draganic D, McCabe K, Richards J, Nasir A, Allen J, et al. Australian Schizophrenia Research Bank: a database of comprehensive clinical, endophenotypic and genetic data for aetiological studies of schizophrenia. Aust N Z J Psychiatry. 2010;44:1029–1035.
